# Supplementary material for: Measuring and valuing spillover effects in caregivers and families: A scoping review
Source: PLoS One. 2026 Mar 24;21(3):e0337253. doi: 10.1371/journal.pone.0337253 (PMC13012466; doi:10.1371/journal.pone.0337253)
Supplement: S1 File — (DOCX) [file pone.0337253.s001.docx]

# **S1 File: Scoping Review of Spillover Effects in Families Protocol**

## **1. Review Title**

Measuring and valuing spillover effects of injury and illness in families: A scoping review.

## **2. Anticipated start date**

7 July 2021.

## **3. Anticipated completion date**

31 October 2025.

## **4. Review Questions**

### *Primary*

1. What methodologies have been used to measure and value spillover effects in caregivers and families?
   1. What are the advantages and disadvantages of each approach?
   2. Have these approaches been applied to veteran, first responder, or FIFO/DIDO families?
   3. If yes, what were the findings?

### *Secondary*

1. What mechanisms are proposed to produce spillover effects in caregivers and families?
   1. What is the conceptual and empirical support for each mechanism?
   2. How have these latent variables been measured?
   3. Are these measures psychometrically sound?
2. What factors are proposed to mediate and moderate spillover effects in caregivers and families?
   1. What is the conceptual and empirical support for each mediator/moderator?
   2. How have these latent variables been measured?
   3. Are these measures psychometrically sound?
3. What are the proposed relationships between the mechanisms, mediators, and moderators?
   1. Have these relationships been formalised as theoretical models?
   2. Have the theoretical models been tested?
   3. What is the conceptual and empirical support for each model?

## **5. Searches**

### *Databases*

- PubMed
- APA PsycInfo via EBSCOhost (all databases: APA PsycInfo, APA PsycArticles, APA PsycBooks); originally searched APA PyscInfo via APA PsycNet on 16 February 2022.
- CINAHL Complete via EBSCOhost
- EconLit via EBSCOhost

### *Search restrictions*

- Language: English.
- Supplementary searching will be limited to hand searching within the reference lists of extracted articles following full-text screening.

## **6. Condition or Domain being studied**

Family health with a specific focus on methods for measuring and valuing spillover effects of injury and illness in caregivers and families; where “Family health” was defined using the PubMed Medical Subject Heading (MeSH): “The health status of the family as a unit including the impact of the health of one member of the family on the family as a unit and on individual family members; also, the impact of family organisation or disorganisation on the health status of its members”.^1^

## **7. Participants**

*Inclusion criteria:*

- Human studies.
- Caregiving and non-caregiving family members (both adults and children) with a family member who is injured or ill.

*Exclusion criteria:*

- Animal studies.

## **8. Concept**

*Inclusion criteria:*

- Non-monetary spillover effects (e.g., health-related quality of life, wellbeing, productivity, educational outcomes, occupational outcomes, labour force participation, social participation) due to injury or illness in a family member.
- Monetary spillover effects (e.g., financial costs of informal care) due to injury or illness in a family member.

*Exclusion criteria:*

- Non-monetary and monetary spillover effects due to death of a family member.
- Zoonotic spillover effects.

## **9. Context**

There will be no restriction for study inclusion based on location or setting.

## **10. Types of Studies to be included**

### *Inclusion criteria*

- Peer-reviewed, quantitative, qualitative, or mixed-methods studies examining spillover effects in families (i.e., the effect of one family members’ health status on the family unit and/or on individual family members; including the impact of family dis/organisation on the health status of family members).

### *Exclusion criteria*

- Grey literature.
- Certain publication types (e.g., comments, editorials, letters).

## **11. Main outcomes**

- Methodologies that have been used to measure and value spillover effects in caregivers and families.

## **12. Additional outcomes**

- Mechanisms, mediators, and moderators of spillover effects in caregivers and families.
- Conceptual and theoretical models of spillover effects in caregivers and families.

## **13. Data extraction (selection and coding)**

The data charting process will involve the following phases:

- A standardised title and abstract form will be developed and piloted by four reviewers on the same 30-50 abstracts to calibrate and test the review form. Two reviewers will independently screen at least 20% of the titles and abstracts, and a third reviewer will resolve any conflicts. Two reviewers will screen the remaining abstracts; and a third reviewer will resolve any conflicts.
- A standardised full-text form will be developed and piloted by four reviewers on the same 5-10 full-text articles to calibrate and test the review form. Two reviewers will screen all included full-text articles; and a third reviewer will resolve any conflicts.
- Two reviewers will extract data from the studies using a piloted form with a set of required data items. A third reviewer will check the accuracy and completeness of the extracted data.

The systematic review software that will be used to facilitate review management, and ensure a fully transparent review process, include EndNote (for citation management) and Covidence (for screening).

## **14. Risk of bias (quality) assessment**

Not applicable.

## **15. Strategy for data synthesis**

Extracted data will be synthesised into five categories of information on (i) measurement of spillover effects; (ii) methods comparisons; (iii) applications of a valuation method; (iv) instrument comparisons; and (v) mechanisms, mediators, or moderators of spillover effects.

## **16. Analysis of subgroups or subsets**

Given the potential breadth and heterogeneity of the returned articles, categories will not be pre-specified. If a sufficient number of similar studies are returned, findings may be discussed by study type (e.g., quantitative, qualitative, and mixed-methods), approach (quality of life [QALYs] vs. burden of disease [DALYs]), methodology (e.g., direct and indirect elicitation techniques), demographics (e.g., age, gender, ethnic background), and geographic region. In addition, sub analyses may explore differences according to the specific populations of interest (i.e., military, first responder, and FIFO/DIDO families).

## **17. Type and method of review**

### Scoping review

## **18. Language**

English.

## **19. Country**

Australia.

## **20. Other registration details**

Nil.

## **21. Reference and/or URL for published protocol**

Not applicable.

## **22. Dissemination plans**

In addition to producing reports for the funders of the review, one or more manuscripts will be submitted to peer-reviewed journals in an appropriate field of study. Furthermore, should the review findings warrant a change in practice, a one-page summary report will be prepared and distributed to interested parties.

## **23. Keywords**

scoping review; family health; spillover; methodology; family demands; filial responsibilities; caregiver burden; health-related quality of life; cost-utility analysis; cost of illness; burden of disease; utility.

## **24. Details of any existing review of the same topic by the same authors**

Not applicable.

## **25. Any additional information**

The scoping review will employ the protocol specified in the Preferred Reporting Items for Systematic Reviews and Meta-Analyses (PRISMA) Extension for Scoping Reviews (PRISMA-ScR).^2^ The search strategy will be specified according to the recent extension to the Preferred Reporting Items for Systematic Reviews and Meta-Analyses (PRISMA-S) statement.^3^

The search strategy for the scoping review was developed in consultation with four Liaison Librarians (The University of Queensland). We wish to acknowledge Miranda Newell, Marcos Riba, and David Honeyman for their contributions to the search strategy. We wish to acknowledge Kim Edmunds for her contributions to the search strategy and comments on an earlier version of the protocol. The final search strategy (February 2022) was developed in consultation with Natalie Barker. Lars Eriksson, a Senior Liaison Librarian, peer-reviewed the search strategy. On 16 February 2022, Natalie Barker translated the search strategy, conducted the literature searches for the four databases, generated the EndNote library, de-duplicated the retrieved literature, and uploaded the citations to Covidence for review. The updated literature search (March 2023) was developed in consultation with Greta Vos. Greta Vos translated the APA PsycNet search strategy for the APA PsycInfo database via ESBCOhost, arranged the peer review of the translation, updated the literature searches for the four databases, and generated the EndNote library. Angela Maguire de-duplicated the retrieved literature in EndNote, and uploaded the citations to Covidence for review. The database searches were last executed on 30 April 2025.

**References**

**1.** National Library of Medicine. Family Health. In: PubMed, ed. *Medical Subject Heading (MeSH)*. Online: National Centre for Biotechnology Information; 2024.

**2.** Tricco AC, Lillie E, Zarin W, et al. PRISMA Extension for Scoping Reviews (PRISMA-ScR): Checklist and explanation. *Ann Intern Med.* Oct 2 2018;169(7):467-473.

**3.** Rethlefsen ML, Kirtley S, Waffenschmidt S, et al. PRISMA-S: An extension to the PRISMA Statement for Reporting Literature Searches in Systematic Reviews. *Systematic Reviews.* 2021/01/26 2021;10(1):39.
