# Supplementary material for: Measuring and valuing spillover effects in caregivers and families: A scoping review
Source: PLoS One. 2026 Mar 24;21(3):e0337253. doi: 10.1371/journal.pone.0337253 (PMC13012466; doi:10.1371/journal.pone.0337253)
Supplement: S3 File — (DOCX) [file pone.0337253.s003.docx]

**S3 File: Search strategy**

The search combined terms relevant to three key concepts: (spillover effects in caregivers and families) **AND** (health economics studies, methods, analyses, and direct and indirect elicitation techniques) **AND** (values, costs, measures, instruments, and outcomes).

**Database searches**

- A search strategy was developed for the PubMed database, including relevant terms from the search strategy reported by Wittenberg et al. (2019)^1^, and then translated for the following three databases: APA PsycInfo, CINAHL Complete, and EconLit.
- All records were exported to an EndNote Library and duplicates removed in two stages:
  - EndNote automatic function;
  - Additional duplicates were then manually identified in EndNote using a title sort and digital object identifier (doi) searches as required.
- Dates searched:
  - Original searches run on 16 February 2022
  - Updated searches run on 20 March 2023
  - Searches last executed on 30 April 2025

**Search limits**

- Publication types:
  - The journal article filter was manually applied for the EBSCO database searches (APA PsycInfo; CINAHL Complete; EconLit);
  - The PubMed search strategy limits to relevant publication types using the following component of the search string: **NOT (**"Comment" [Publication Type] OR "Editorial" [Publication Type] OR "Letter" [Publication Type]**)**.
- Human studies:
  - The PubMed search strategy excludes animal studies using the search string:
    **NOT (**"Animals"[Mesh] NOT "Humans"[Mesh]**)**.
  - The CINAHL Complete search strategy excludes animal studies using the search string: **NOT** **(**(MH "Animals+" OR MH "Animal Studies") NOT MH "Human")
- English language:
  - The PubMed search strategy limits to English language studies using the search string: **AND (**eng[la] OR und[la]**)**.
  - The EBSCO database searches limit to English language studies using the search string: **AND (**LA English**)**

**Search strategy for PubMed**

Includes MeSH

**(**"Family Health"[Mesh] OR "spillover"[tiab] OR "spillovers"[tiab] OR "spill over"[tiab] OR "spill overs"[tiab] OR "caring externalities"[tiab] OR "family health"[tiab] OR "family burden"[tiab] OR "caregiver burden"[tiab] OR "caregiver burdens"[tiab] OR "care giver burdens"[tiab] OR "care giver burden"[tiab] OR "carer burden"[tiab] OR "carer burdens"[tiab] OR "filial responsibility"[tiab] OR "filial responsibilities"[tiab] OR "family demands"[tiab] OR "parentification"[tiab] OR "infantilisation"[tiab] OR "infantilization"[tiab] OR "informal care"[tiab]**) AND (**"Cost of Illness"[Mesh] OR "Cost-Benefit Analysis"[Mesh] OR "Quality-Adjusted Life Years"[Mesh] OR "Disability-Adjusted Life Years"[Mesh] OR "cost utility analysis"[tiab] OR "disutility"[tiab] OR "preference based"[tiab] OR "hrqol"[tiab] OR "quality adjusted life year"[tiab] OR "quality adjusted life years"[tiab] OR "qalys"[tiab] OR "disability adjusted life years"[tiab] OR "dalys"[tiab] OR "SF 6D"[tiab] OR "SF6D"[tiab] OR "EQ 5D"[tiab] OR "EQ5D"[tiab] OR "euroqol"[tiab] OR "carerqol"[tiab] OR "carer experience scale"[tiab] OR "health utilities index"[tiab] OR "quality of wellbeing scale"[tiab] OR "quality of well being scale"[tiab] OR "assessment of quality of life"[tiab] OR "AQOL"[tiab] OR "standard gamble"[tiab] OR "time tradeoff"[tiab] OR "time trade off"[tiab] OR "visual analog"[tiab] OR "visual analogue"[tiab] OR "CHU 9D"[tiab] OR "preference weight*"[tiab] OR "eqvas"[tiab] OR "preference score*"[tiab] OR "economic evaluation"[tiab] OR "economic evaluations"[tiab] OR "economic outcomes"[tiab] OR "economic outcome"[tiab] OR "cost effectiveness"[tiab] OR "illness cost"[tiab] OR "illness costs"[tiab] OR "cost of sickness"[tiab] OR "sickness costs"[tiab] OR "sickness cost"[tiab] OR "burden of illness"[tiab] OR "illness burden"[tiab] OR "illness burdens"[tiab] OR "disease burden"[tiab] OR "disease burdens"[tiab] OR "costs of disease"[tiab] OR "disease cost"[tiab] OR "disease costs"[tiab] OR "burden of disease"[tiab] OR "burden of diseases"[tiab] OR "cost of disease"[tiab] OR "monetary valuation"[tiab] OR (("Quality of Life"[Mesh] OR "quality of life"[tiab]) AND ("Economics"[Mesh] OR "economic*"[tiab]))**)** **AND (**"value"[tiab] OR "values"[tiab] OR "valuation"[tiab] OR "cost"[tiab] OR "costs"[tiab] OR "costing"[tiab] OR "costings"[tiab] OR "measure"[tiab] OR "measures"[tiab] OR "measurement"[tiab] OR "measurements"[tiab] OR "measuring"[tiab] OR "measured"[tiab] OR "evaluation"[tiab] OR "evaluations"[tiab] OR "outcome"[tiab] OR "outcomes"[tiab] OR "instrument"[tiab] OR "instruments"[tiab]**) NOT (**"Comment" [Publication Type] OR "Editorial" [Publication Type] OR "Letter" [Publication Type]**) NOT (**"Animals"[Mesh] NOT "Humans"[Mesh]**) AND (**eng[la] OR und[la]**)**

**Search strategy for APA PsycInfo (EBSCOhost); APA PsycInfo originally searched via APA PsycNet on 16 February 2022**

Includes APA Thesaurus terms

**(** TI (("spillover" OR "spillovers" OR "spill over" OR "spill overs" OR "caring externalities" OR "family health" OR "family burden" OR "caregiver burden" OR "caregiver burdens" OR "care giver burdens" OR "care giver burden" OR "carer burden" OR "carer burdens" OR "filial responsibility" OR "filial responsibilities" OR "family demands" OR "parentification" OR "infantilisation" OR "infantilization" OR "informal care")) OR AB (("spillover" OR "spillovers" OR "spill over" OR "spill overs" OR "caring externalities" OR "family health" OR "family burden" OR "caregiver burden" OR "caregiver burdens" OR "care giver burdens" OR "care giver burden" OR "carer burden" OR "carer burdens" OR "filial responsibility" OR "filial responsibilities" OR "family demands" OR "parentification" OR "infantilisation" OR "infantilization" OR "informal care")) **) AND** **(** (DE "Quality of Life Measures") OR TI (("cost utility analysis" OR "disutility" OR "preference based" OR "hrqol" OR "quality adjusted life year" OR "quality adjusted life years" OR "qalys" OR "disability adjusted life years" OR "dalys" OR "SF 6D" OR "SF6D" OR "EQ 5D" OR "EQ5D" OR "euroqol" OR "carerqol" OR "carer experience scale" OR "health utilities index" OR "quality of wellbeing scale" OR "quality of well being scale" OR "assessment of quality of life" OR "AQOL" OR "standard gamble" OR "time tradeoff" OR "time trade off" OR "visual analog" OR "visual analogue" OR "CHU 9D" OR "preference weight*" OR "eqvas" OR "preference score*" OR "economic evaluation" OR "economic evaluations" OR "economic outcomes" OR "economic outcome" OR "cost effectiveness" OR "illness cost" OR "illness costs" OR "cost of sickness" OR "sickness costs" OR "sickness cost" OR "burden of illness" OR "illness burden" OR "illness burdens" OR "disease burden" OR "disease burdens" OR "costs of disease" OR "disease cost" OR "disease costs" OR "burden of disease" OR "burden of diseases" OR "cost of disease" OR "monetary valuation")) OR AB (("cost utility analysis" OR "disutility" OR "preference based" OR "hrqol" OR "quality adjusted life year" OR "quality adjusted life years" OR "qalys" OR "disability adjusted life years" OR "dalys" OR "SF 6D" OR "SF6D" OR "EQ 5D" OR "EQ5D" OR "euroqol" OR "carerqol" OR "carer experience scale" OR "health utilities index" OR "quality of wellbeing scale" OR "quality of well being scale" OR "assessment of quality of life" OR "AQOL" OR "standard gamble" OR "time tradeoff" OR "time trade off" OR "visual analog" OR "visual analogue" OR "CHU 9D" OR "preference weight*" OR "eqvas" OR "preference score*" OR "economic evaluation" OR "economic evaluations" OR "economic outcomes" OR "economic outcome" OR "cost effectiveness" OR "illness cost" OR "illness costs" OR "cost of sickness" OR "sickness costs" OR "sickness cost" OR "burden of illness" OR "illness burden" OR "illness burdens" OR "disease burden" OR "disease burdens" OR "costs of disease" OR "disease cost" OR "disease costs" OR "burden of disease" OR "burden of diseases" OR "cost of disease" OR "monetary valuation")) OR ((DE "Quality of Life" OR DE "Health Related Quality of Life") OR TI ("quality of life") OR AB ("quality of life") AND (DE "Economics" OR DE "Health Care Economics") OR TI ("economic*") OR AB (“economic*”)) **)** **AND** **(** TI (("value" OR "values" OR "valuation" OR "cost" OR "costs" OR "costing" OR "costings" OR "measure" OR "measures" OR "measurement" OR "measurements" OR "measuring" OR "measured" OR "evaluation" OR "evaluations" OR "outcome" OR "outcomes" OR "instrument" OR "instruments")) OR AB (("value" OR "values" OR "valuation" OR "cost" OR "costs" OR "costing" OR "costings" OR "measure" OR "measures" OR "measurement" OR "measurements" OR "measuring" OR "measured" OR "evaluation" OR "evaluations" OR "outcome" OR "outcomes" OR "instrument" OR "instruments")) **) AND (**LA English**)**

**Search strategy for CINAHL Complete (EBSCOhost)**

Includes CINAHL Subject Headings

**(**MH "Family Health" OR TI("spillover" OR "spillovers" OR "spill over" OR "spill overs" OR "caring externalities" OR "family health" OR "family burden" OR "caregiver burden" OR "caregiver burdens" OR "care giver burdens" OR "care giver burden" OR "carer burden" OR "carer burdens" OR "filial responsibility" OR "filial responsibilities" OR "family demands" OR "parentification" OR "infantilisation" OR "infantilization" OR "informal care") OR AB("spillover" OR "spillovers" OR "spill over" OR "spill overs" OR "caring externalities" OR "family health" OR "family burden" OR "caregiver burden" OR "caregiver burdens" OR "care giver burdens" OR "care giver burden" OR "carer burden" OR "carer burdens" OR "filial responsibility" OR "filial responsibilities" OR "family demands" OR "parentification" OR "infantilisation" OR "infantilization" OR "informal care")**) AND (**MH "Economic Aspects of Illness" OR MH "Cost Benefit Analysis" OR MH "Disability-Adjusted Life Years" OR MH "Quality-Adjusted Life Years" OR TI("cost utility analysis" OR "disutility" OR "preference based" OR "hrqol" OR "quality adjusted life year" OR "quality adjusted life years" OR "qalys" OR "disability adjusted life years" OR "dalys" OR "SF 6D" OR "SF6D" OR "EQ 5D" OR "EQ5D" OR "euroqol" OR "carerqol" OR "carer experience scale" OR "health utilities index" OR "quality of wellbeing scale" OR "quality of well being scale" OR "assessment of quality of life" OR "AQOL" OR "standard gamble" OR "time tradeoff" OR "time trade off" OR "visual analog" OR "visual analogue" OR "CHU 9D" OR "preference weight*" OR "eqvas" OR "preference score*" OR "economic evaluation" OR "economic evaluations" OR "economic outcomes" OR "economic outcome" OR "cost effectiveness" OR "illness cost" OR "illness costs" OR "cost of sickness" OR "sickness costs" OR "sickness cost" OR "burden of illness" OR "illness burden" OR "illness burdens" OR "disease burden" OR "disease burdens" OR "costs of disease" OR "disease cost" OR "disease costs" OR "burden of disease" OR "burden of diseases" OR "cost of disease" OR "monetary valuation") OR AB("cost utility analysis" OR "disutility" OR "preference based" OR "hrqol" OR "quality adjusted life year" OR "quality adjusted life years" OR "qalys" OR "disability adjusted life years" OR "dalys" OR "SF 6D" OR "SF6D" OR "EQ 5D" OR "EQ5D" OR "euroqol" OR "carerqol" OR "carer experience scale" OR "health utilities index" OR "quality of wellbeing scale" OR "quality of well being scale" OR "assessment of quality of life" OR "AQOL" OR "standard gamble" OR "time tradeoff" OR "time trade off" OR "visual analog" OR "visual analogue" OR "CHU 9D" OR "preference weight*" OR "eqvas" OR "preference score*" OR "economic evaluation" OR "economic evaluations" OR "economic outcomes" OR "economic outcome" OR "cost effectiveness" OR "illness cost" OR "illness costs" OR "cost of sickness" OR "sickness costs" OR "sickness cost" OR "burden of illness" OR "illness burden" OR "illness burdens" OR "disease burden" OR "disease burdens" OR "costs of disease" OR "disease cost" OR "disease costs" OR "burden of disease" OR "burden of diseases" OR "cost of disease" OR "monetary valuation") OR ((MH "Quality of Life+" OR TI("quality of life") OR AB("quality of life")) AND (MH "Economics+" OR TI("economic*") OR AB("economic*")))**)** **AND (**TI("value" OR "values" OR "valuation" OR "cost" OR "costs" OR "costing" OR "costings" OR "measure" OR "measures" OR "measurement" OR "measurements" OR "measuring" OR "measured" OR "evaluation" OR "evaluations" OR "outcome" OR "outcomes" OR "instrument" OR "instruments") OR AB("value" OR "values" OR "valuation" OR "cost" OR "costs" OR "costing" OR "costings" OR "measure" OR "measures" OR "measurement" OR "measurements" OR "measuring" OR "measured" OR "evaluation" OR "evaluations" OR "outcome" OR "outcomes" OR "instrument" OR "instruments")**)** **NOT** **(**(MH "Animals+" OR MH "Animal Studies") NOT MH "Human") **AND (**LA English**)**

**Search strategy for EconLit (EBSCOhost)**

**(**TI("spillover" OR "spillovers" OR "spill over" OR "spill overs" OR "caring externalities" OR "family health" OR "family burden" OR "caregiver burden" OR "caregiver burdens" OR "care giver burdens" OR "care giver burden" OR "carer burden" OR "carer burdens" OR "filial responsibility" OR "filial responsibilities" OR "family demands" OR "parentification" OR "infantilisation" OR "infantilization" OR "informal care") OR AB("spillover" OR "spillovers" OR "spill over" OR "spill overs" OR "caring externalities" OR "family health" OR "family burden" OR "caregiver burden" OR "caregiver burdens" OR "care giver burdens" OR "care giver burden" OR "carer burden" OR "carer burdens" OR "filial responsibility" OR "filial responsibilities" OR "family demands" OR "parentification" OR "infantilisation" OR "infantilization" OR "informal care")**) AND (**TI("cost utility analysis" OR "disutility" OR "preference based" OR "hrqol" OR "quality adjusted life year" OR "quality adjusted life years" OR "qalys" OR "disability adjusted life years" OR "dalys" OR "SF 6D" OR "SF6D" OR "EQ 5D" OR "EQ5D" OR "euroqol" OR "carerqol" OR "carer experience scale" OR "health utilities index" OR "quality of wellbeing scale" OR "quality of well being scale" OR "assessment of quality of life" OR "AQOL" OR "standard gamble" OR "time tradeoff" OR "time trade off" OR "visual analog" OR "visual analogue" OR "CHU 9D" OR "preference weight*" OR "eqvas" OR "preference score*" OR "economic evaluation" OR "economic evaluations" OR "economic outcomes" OR "economic outcome" OR "cost effectiveness" OR "illness cost" OR "illness costs" OR "cost of sickness" OR "sickness costs" OR "sickness cost" OR "burden of illness" OR "illness burden" OR "illness burdens" OR "disease burden" OR "disease burdens" OR "costs of disease" OR "disease cost" OR "disease costs" OR "burden of disease" OR "burden of diseases" OR "cost of disease" OR "monetary valuation") OR AB("cost utility analysis" OR "disutility" OR "preference based" OR "hrqol" OR "quality adjusted life year" OR "quality adjusted life years" OR "qalys" OR "disability adjusted life years" OR "dalys" OR "SF 6D" OR "SF6D" OR "EQ 5D" OR "EQ5D" OR "euroqol" OR "carerqol" OR "carer experience scale" OR "health utilities index" OR "quality of wellbeing scale" OR "quality of well being scale" OR "assessment of quality of life" OR "AQOL" OR "standard gamble" OR "time tradeoff" OR "time trade off" OR "visual analog" OR "visual analogue" OR "CHU 9D" OR "preference weight*" OR "eqvas" OR "preference score*" OR "economic evaluation" OR "economic evaluations" OR "economic outcomes" OR "economic outcome" OR "cost effectiveness" OR "illness cost" OR "illness costs" OR "cost of sickness" OR "sickness costs" OR "sickness cost" OR "burden of illness" OR "illness burden" OR "illness burdens" OR "disease burden" OR "disease burdens" OR "costs of disease" OR "disease cost" OR "disease costs" OR "burden of disease" OR "burden of diseases" OR "cost of disease" OR "monetary valuation") OR ((TI("quality of life") OR AB("quality of life")) AND (TI("economic*") OR AB("economic*")))**)** **AND (**TI("value" OR "values" OR "valuation" OR "cost" OR "costs" OR "costing" OR "costings" OR "measure" OR "measures" OR "measurement" OR "measurements" OR "measuring" OR "measured" OR "evaluation" OR "evaluations" OR "outcome" OR "outcomes" OR "instrument" OR "instruments") OR AB("value" OR "values" OR "valuation" OR "cost" OR "costs" OR "costing" OR "costings" OR "measure" OR "measures" OR "measurement" OR "measurements" OR "measuring" OR "measured" OR "evaluation" OR "evaluations" OR "outcome" OR "outcomes" OR "instrument" OR "instruments")**) AND (**LA English**)**

**References**

**1.** Wittenberg E, James LP, Prosser LA. Spillover effects on caregivers' and family members' utility: A systematic review of the literature. *Pharmacoeconomics.* Apr 2019;37(4):475-499.
