# Supplementary material for: Measuring and valuing spillover effects in caregivers and families: A scoping review
Source: PLoS One. 2026 Mar 24;21(3):e0337253. doi: 10.1371/journal.pone.0337253 (PMC13012466; doi:10.1371/journal.pone.0337253)
Supplement: S4 File — Table S4.2. Psychometric and clinimetric comparison of instruments for measuring spillover effects. Table S4.3. Time Trade-Off studies for utility elicitation. Table S4.4. Mediators and moderators of spillover effects. Table S4.5. Factors acting as both mediators and moderators. (DOCX) [file pone.0337253.s004.docx]

# **S4 File: Supplemental Tables**

**Table S4.1. Impact on reported ICERs of including family/caregiver spillover effects and broader value elements**

| **Study** | **Care recipient condition** | **Spillover domains** | **Key findings on ICER impact** |
| --- | --- | --- | --- |
| **Including spillovers decreases ICERs (Interventions appeared more cost-effective)** | | | |
| Al-Janabi-2021 (1) | Multiple diseases (dementia, mental health, stroke) | Informal care time  HRQOL/Wellbeing impact  Employment | Stakeholder participants predicted interventions affected multiple carer domains; 80–81% supported considering carer effects in research studies, funding decisions, and patient decision-making. |
| Angelini-2023 (2) | Cancer | HRQOL/Wellbeing impact | Significant negative health and well-being spillovers on partners; inclusion highlights fuller burden and may improve perceived intervention cost-effectiveness. |
| Aranda-Reneo-2021 (3) | Rare diseases | Informal care costs  Productivity loss | Considering societal costs changed 3 of 26 economic evaluation estimates from cost-effective to dominant, but modified the authors’ conclusion in one study. |
| Duevel-2020 (4) | Depression | Informal care cost  HRQOL/Wellbeing impact  Productivity loss  Broader value elements | Including societal costs changed the cost-effectiveness quadrant in 24% of cases (5% became cost-effective, 4% became not cost-effective). The chosen perspective (e.g., societal vs. healthcare) can significantly influence cost-utility outcomes. |
| Lavelle-2019 (5) | Childhood illness | Informal care time  Informal care cost  HRQOL/Wellbeing impact  Productivity loss | 72% cost-utility analyses included family spillover costs or health effects. Including spillover reduced ICERs by 31% (~$40,000/QALY); 19/43 ICER pairs (which were compared with and without spillover) crossed cost-effectiveness thresholds. |
| Leech-2023 (6) | Multiple diseases | Informal care time  Informal care cost  HRQOL/Wellbeing impact  Productivity loss | Including family spillovers decreased ICERs by 31–42%, sometimes crossing health technology assessment thresholds and affecting reimbursement decisions. |
| Lin-2019 (7) | Dementia, Alzheimer’s disease | Informal care cost  HRQOL/Wellbeing impact  Productivity loss | 70% of Alzheimer’s cost-utility analyses included spillovers; including these spillover outcomes generally made ICERs more favourable (45%) or kept interventions cost-saving (39%). In 11 cases (33%), adding spillovers changed the ICER to cross a common threshold. |
| Ma-2022 (8) | HPV infection; Early-stage Hodgkin’s lymphoma (ESHL) | Informal care time  HRQOL/Wellbeing impact  Productivity loss | HPV vaccination became cost saving when productivity included. For combined modality therapy in ESHL patients, including all value elements slightly reduced its net monetary benefit despite increased near-term health gains. |
| Pennington-2020 (9) | Multiple sclerosis | HRQOL/Wellbeing impact | Out of 414 technology appraisals, only 12 (3%) included carer HRQOL in cost-utility analyses; 4 of 8 highly specialised technologies (50%) included it. Including carer HRQOL consistently increased incremental QALYs and lowered ICERs. |
| Ride-2018 (10) | Postnatal depression | Health and social care costs  HRQOL/Wellbeing impact  Productivity loss | Broader analyses that accounted for longer-term impacts and family effects often showed interventions to be much more cost-effective than narrower analyses focused only on the mother's short-term health outcomes. |
| Rodriguez-Sanchez-2021 (11) | Diabetes | Informal care time  Informal care cost  Productivity loss | Including social costs changed conclusions in 17% of estimations; some shifted from not cost-effective to dominant. |
| Rodríguez-Sánchez-2023 (12) | Multiple sclerosis | Informal care cost Productivity loss | Including social costs changed 10 conclusions; 6 shifted from not recommended to recommended; 5 from cost-effective to dominant. |
| Yuasa-2022 (13) | Chronic immune-mediated disorders | Productivity loss | 49 studies included productivity losses/gains. Including productivity costs improved ICERs in 57% of studies; 24.5% saw no major impact; including productivity improved cost-effectiveness results. |
| **Including spillovers increases ICERs (Interventions appeared less cost-effective)** | | | |
| Bhadhuri-2019 (14) | COPD | HRQOL/Wellbeing impact | Including household member QALYs slightly increased ICERs (£10,271 → £10,991; up to £12,913 with household healthcare costs). Minimal impact on cost-effectiveness. |
| de Groot-2023 (15) | Old age (individuals older than 50) | Informal care time  Informal care cost | Including informal care costs changed ICERs by €200–€17,700 per QALY; sometimes altered intervention ranking. |
| Mott-2023 (16) | Multiple diseases | HRQOL/Wellbeing impact | “Carer QALY trap”: Including caregiver disutility can reduce net QALYs, making life-extending treatments less cost-effective. |
| Shafrin-2021 (17) | Multiple diseases | Broader value elements | More value elements cited in ICER reports correlated with higher ICERs. |
| **Unspecified direction of change** | | | |
| Breslau-2023 (18) | Multiple diseases | Broader value elements | Most health technology assessment guidelines mention few societal elements; inclusion could alter evaluations indirectly. |
| Canadian Agency for Drugs and Technologies in Health-2024 (19) | Childhood illness | HRQOL/Wellbeing impact | Rarely quantified in models; limited ICER impact but recognised importance. |
| Drost-2017 (20) | Multiple diseases | Intersectoral costs and benefits | Including broader costs (like productivity and informal care) could significantly change the ICER outcomes. |
| Park-2022 (21) | Alzheimer’s disease | Broader value elements | Estimated family spillover costs $57 billion in 2020; ignoring underestimates societal burden. |

*COPD, Chronic obstructive pulmonary disease; ESHL, Early-stage Hodgkin’s lymphoma; HPV, Human papillomavirus; HRQOL, Health-related quality of life; ICER, Incremental cost-effectiveness ratio; QALY, Quality-adjusted life year.*

**Table S4.2. Psychometric and clinimetric comparison of instruments for measuring spillover effects.**

| **Study** | **Care recipient condition** | **Spillover domain** | **Instruments being compared** | **Aspects of instrument comparisons** | **Key findings** |
| --- | --- | --- | --- | --- | --- |
| **Comparisons among carer-specific measures** | | | | | |
| Bucholc-2023 (22) | Multiple diseases | HRQOL/Wellbeing impact | - ASCOT-Carer - CarerQOL-7D - CES | - Construct validity  - Missing aspects and ranked the importance of instrument domains | All three instruments were considered relevant, with domains viewed as important by carers. Some aspects that carers perceived as missing were in fact covered, highlighting differences in interpretation. They less directly captured the impact of care recipients’ health and well-being on carer QOL. |
| Eagleson-2023 (23) | Congenital heart disease (CHD) | Caregiver burden  HRQOL/Wellbeing impact | 97 instruments, including psychological distress scales (e.g. BDI, STAI), parenting stress indices, QOL measures (e.g. SF-36, PedsQL), and family functioning tools (e.g. IOFS). | - Content validity - Internal consistency - Test-retest reliability - Responsiveness (longitudinal validity) | A wide range of instruments were used, most commonly assessing psychological functioning/distress, coping, and QOL. Psychometric quality was variable: 67% demonstrated content validity, 39% showed adequate internal consistency, 4% showed test–retest reliability, and 9% showed responsiveness. Most tools were generic and self-reported, with only one CHD-specific instrument, highlighting the need for stronger psychometric reporting, a toolkit approach, and development of comprehensive CHD-specific family measures. |
| Engel-2020 (24) | Multiple diseases | HRQOL/Wellbeing impact | - ASCOT-Carer - CES - CarerQOL | - Construct validity  - Overlapping or differing domains | The three measures capture distinct constructs and are not interchangeable. A five-factor model emerged, covering general QOL outside caring, problems due to caring, fulfilment from caring, social support, and relationship with the care recipient. ASCOT-Carer and CarerQOL loaded mainly onto separate factors, with some overlap between CarerQOL, CES, and shared domains, but each remained distinct. |
| Gonçalves-Pereira-2017 (25) | Schizophrenia | Caregiver burden  HRQOL/Wellbeing impact | - ZBI  - IEQ  - GHQ-28 - CES-D | - Internal consistency - Convergent validity  - Discriminative validity (via ROC and AUC against GHQ-28 and CES-D) - Sensitivity to change (standardised effect size) | Both instruments are reliable and valid for assessing caregiver burden, showing strong internal consistency (α = 0.91 and 0.86, respectively) and moderate to high convergent validity, with correlations between related subscales ranging from 0.66 to 0.69. Both also demonstrated comparable discriminative validity against GHQ and CES-D cut-offs, with no significant differences in AUC values. |
| Hamidou-2017 (26) | Cancer | HRQOL/Wellbeing impact | - SEIQOL-DW - EORTC QLQ-C30 - CarGOQOL - SF-36 | - Validity - Overlap | The SEIQOL index showed significant correlations with condition-specific (EORTC QLQ-C30, CarGOQoL) and generic (SF-36) measures for both patients and caregivers, particularly for psychological, social, and mental health domains. Caregivers often reported lower family-related QOL but prioritised health, reflecting spillover effects from the patient’s illness. Patient and caregiver QOL scores were interrelated, highlighting the impact of patient health on caregiver wellbeing. |
| Kawakita-2024 (27) | Multiple diseases | HRQOL/Wellbeing impact  Skills and perspectives, psychological and physical resources, social-capital resources, flexibility, and material resources | - Work-Family Enrichment Scale for Parent Nurses (WFES-PN and FWES-PN) - Japanese Work-Family Enrichment Scale (J-WFES) - Positive Spillover Scale | - Validity - Reliability - Internal consistency - Factor analysis - Test-retest reliability | The WFES-PN demonstrated strong reliability and validity, with five-factor structures identified for both work-to-family and family-to-work directions (Cronbach’s α = 0.79–0.91). Correlations with existing J-WFES and Positive Spillover Scales supported criterion-related validity. The scale effectively measures positive aspects of work and family roles among female parent nurses. |
| Kudra-2017 (28) | Multiple diseases (Parkinson’s disease, heart failure, multiple sclerosis, COPD, cancer) | Informal care time  Informal care cost  Caregiver burden  HRQOL/Wellbeing impact | - ZBI  - CRA - Other tools, e.g., CDS, BSFC | - Construct validity  - Applicability to specific diseases - Coverage of burden domains | The instruments are the most commonly used tools to assess caregiver burden across chronic illnesses such as Parkinson’s, heart failure, multiple sclerosis, and COPD. ZBI focuses on overall burden, while CRA assesses both negative impacts (e.g., schedule disruption, finances, health) and positive aspects like caregiver self-esteem. Burden varies by disease and caregiving context, and some tools lack full validation for certain conditions, highlighting the need for improved instruments. |
| Kuharic-2025 (29) | Multiple diseases (chronic health conditions, disabilities, aging) | Caregiver burden | -CARE-2B (included 2 subscales CR-SPB and Proxy-CB)  - Self-Perceived Burden (SPB)  - EQ-5D-5L  - EQ-HWB (completed by care recipients) - CarerQOL-7D and ASCOT-Carer (completed by caregivers) | - Construct validity (through EFA and CFA) - Convergent validity - Item discrimination - Difficulty parameters - Information coverage using item response theory analysis - Distinctiveness and overlap of the CARE-2B subscales with other health and social care constructs | The CARE-2B Scale distinguishes care recipients’ self-perceived burden (CR-SPB) from their perception of caregiver burden (Proxy-CB). CR-SPB aligns with the recipient’s health, while Proxy-CB aligns with caregiver-reported burden. The scale is valid, reliable, and can help identify needs in caregiving situations when caregivers’ reports are unavailable. |
| McCaffrey-2020 (30) | Multiple diseases (chronic, progressive, or life-limiting illnesses) | HRQOL/Wellbeing impact | - ASCOT-Carer - CES  - CarerQOL - CRA  - EQ-5D-5L  - AQOL-8D | - Feasibility (completion rates) - Reliability (internal consistency, test–retest) - Validity (convergent and discriminative) - Ceiling/floor effects | The ASCOT-Carer showed the highest internal consistency and test-retest reliability compared to CarerQOL and CES. All three instruments demonstrated convergent validity and detected significant associations with informal care situations, though CarerQOL-7D missed sole carer status. ASCOT-Carer had the strongest psychometric performance in this sample. |
| Sampogna-2017 (31) | Dermatological conditions | Caregiver burden HRQOL/Wellbeing impact  A wide range of daily life and social impacts | 9 main instruments in dermatology* | - Validity (conceptual, construct, convergent) - Interpretability (norms, categorisation, minimal clinically important difference) - Reliability (internal consistency, retest reliability) - Structure - Responsiveness - Item bias - Cultural issues (translations, cultural equivalence) - Respondent burden - Administrative burden - Availability of alternative forms | Nine questionnaires assessing how skin conditions impact family and caregiver QOL were identified. Most are new and require further validation, particularly for score interpretation and cross-cultural use. Instruments for paediatric conditions like atopic dermatitis are more common, but widely validated tools for other dermatological conditions are lacking. |
| Tu-2022 (32) | Mental disorders (dementia, Alzheimer's disease) | Caregiver burden | 4 recommended instruments:  - ZBI  - SCB  - CBI  - BSFC  Identified 14 other self-report instruments for dementia caregiver burden | - Reliability (internal consistency, inter-rater reliability) - Validity (face, content, convergent, construct) - Multidimensionality (inclusion of objective and subjective items, explicit subscores/factors) - Expedience (number of items and time needed for administration) | ZBI, SCB, CBI, and BSFC are recommended as reliable and valid self-report tools for measuring dementia caregiver burden, with some providing more targeted assessments. Most studies are cross-sectional and focus on unpaid informal caregivers, underscoring the need for longitudinal research and the inclusion of paid or foreign caregivers. |
| **Comparisons among utility measures** | | | | | |
| Bhadhuri-2017 (33) | Meningitis | HRQOL/Wellbeing impact | - EQ-5D-5L - SF-6D | - Construct validity - Responsiveness - Effect sizes and correlation strengths  - Responsiveness to change in patient health and caregiving hours | Both instruments were valid for capturing HRQOL spillovers in family members. For carers, EQ-5D-5L was more responsive to spillovers from patient health, while SF-6D better reflected spillovers from informal care provision. |
| Brown-2019 (34) | Autism spectrum disorder | HRQOL/Wellbeing impact | - EQ-5D-3L - SF-6D (from SF-12 v2.0) | - Convergent validity - Clinical validity - Discriminative power in detecting caregiver health effects | Both instruments are valid for assessing caregiver spillover effects, with SF-6D showing greater sensitivity to child behavior and well-being. EQ-5D-3L yielded higher average scores but lower discriminative power. SF-6D detected more significant group differences and better captured spillover effects in cost-effectiveness analysis. |
| Ertzgaard-2019 (35) | Nervous system diseases (spinal cord injury/damage ‘SCD’, traumatic brain injury, stroke, multiple sclerosis) | Caregiver burden HRQOL/Wellbeing impact | - Spasticity-specific patient-reported outcome measures† - Generic HRQOL measures - Preference-based measures  - Caregiver burden measures | - Clinical relevance and practicality  - Psychometric properties (reliability, validity, responsiveness) - Strengths and weaknesses - Suitability for economic evaluation and prioritizing healthcare funding - Linguistic availability - Cost of use (license fees) - Ability to detect clinical changes - Acceptability to patients | Two condition-specific (PRISM, SCI-SET), five generic, and four preference-based measures were identified. PRISM and SCI-SET showed strong psychometric support, while SF-36 is useful and can be adapted or converted to SF-6D; no SCD-specific caregiver tools exist, but Caregiver Burden Scale and ZBI are suitable. A combination of condition-specific (PRISM or SCI-SET) and generic (SF-36) measures is recommended for comprehensive assessment. |
| Faraji-2024 (36) | Multiple sclerosis | Caregiver burden  HRQOL/Wellbeing impact | - EQ-5D-3L  - HUI-3 - CarerQOL-7D - CES-D | - Convergent validity - Clinical validity - Discriminative power | EQ-5D-3L and HUI-3 showed strong correlation (*r* = 0.914) and clinical validity for assessing caregiver QOL, with HUI-3 demonstrating better consistency with CarerQOL-7D. Caregiver QOL scores were lower among female, single, lower-educated, non-parent relatives, and unemployed caregivers. HUI-3 is preferred over EQ-5D-3L due to better alignment with CarerQOL-7D. |
| McLoughlin-2020 (37) | Multiple diseases (dementia, stroke, mental illnesses, rheumatoid arthritis) | HRQOL/Wellbeing impact | - CES  - CarerQOL-7D - ASCOT-Carer - EQ-5D-5L - ICECAP-A | - Response rates - Convergent validity - Construct validity - Responsiveness | Carer-specific QOL measures (CES, CarerQOL-7D, ASCOT-Carer) were strongly interrelated, with ICECAP-A also showing strong connections, while EQ-5D-5L was less related. ASCOT-Carer and ICECAP-A better reflected factors influencing carer QOL, and over time, CarerQOL-7D was more responsive to patient health changes, whereas ASCOT-Carer was more responsive to caregiving hours. |
| McLoughlin-2023 (38) | Multiple diseases (dementia, stroke, mental illnesses) | HRQOL/Wellbeing impact | - ASCOT-Carer - CarerQOL-7D - CES - EQ-5D-5L - ICECAP-A | - Content validity - Face validity - Feasibility (ease of use, response issues, language clarity, response scale) | The instruments were generally feasible and understandable, with ASCOT-Carer and CES most preferred, though some measures lacked nuance or missed topics like financial strain, and certain questions caused confusion or emotional difficulty after recent bereavement. |
| Monteiro-2022 (39) | Multiple diseases (cancer, mental illnesses, chronic physical conditions) | HRQOL/Wellbeing impact | - EQ-HWB-S - EQ-5D-5L - SWEMWBS  - ASCOT | - Content coverage (conceptual overlap of dimensions) - Acceptability (item missingness) - Response distributions (frequencies for each item level) - Ceiling/floor effects - Convergent validity  - Discriminative ability | The EQ-HWB-S and EQ-5D-5L showed moderate to strong overlap, but EQ-HWB-S could detect subtle issues like exhaustion even when EQ-5D-5L indicated perfect health. Both measures differentiated well between people with and without health conditions, but only EQ-HWB-S distinguished caregivers from non-caregivers and low versus high caregiver burden, though differences were small. |
| Reed-2017 (40) | Dementia, Alzheimer's disease | Informal care time  Caregiver burden  HRQOL/Wellbeing impact | - EQ-5D - ZBI - RUD | - Construct validity  - Responsiveness/sensitivity to change  - Concurrent associations | The EQ-5D, focusing mainly on physical health, poorly captures the full impact of caregiving for people with Alzheimer's disease. Its overall scores showed weak correlations with ZBI scores and time spent on care, and remained largely unchanged over 18 months despite increasing patient disease severity. In contrast, caregiver burden and time spent caring significantly increased with the patient's disease severity. |
| Vatter-2020 (41) | Multiple diseases (Parkinson's disease, dementia with Lewy bodies, mild cognitive impairment, dementia) | Caregiver burden  HRQOL/Wellbeing impact | - ZBI - Rel.SS  - DRS  - FCR  - BRS  - RSS  - SF-12 - HADS  - EQ-5D-3L | - Psychometric properties (internal consistency, convergent validity, floor and ceiling effects, completion rate, data missingness) - Clinimetric properties (time to administer, ease of scoring, readability, and availability/cost) | 7/9 instruments (ZBI, HADS, SF-12, Rel.SS, EQ-5D, BRS, and RSS) showed good psychometric properties and clinical utility, while two (DRS and FCR) performed poorly. Most measures were quick to complete and generally accessible, though some (SF-12, EQ-5D) were pricier and harder to score. The original development studies for all measures were rated as methodologically inadequate. |
| Zhou-2025 (42) | COVID-19 in children | Caregiver burden HRQOL/Wellbeing impact | - EQ-5D-5L  - EQ-HWB-S  - Other instrments: EQ VAS, OHA, EQ-TIPS, EQ-5D-Y-3L | - Known-groups validity - Test-retest reliability - Responsiveness to health improvement - Distribution of dimension responses | Both EQ-HWB-S and EQ-5D-5L are valid and reliable for measuring parental spillover from child illness. EQ-HWB-S better captures social/emotional impacts and differences related to child illness, caregiving time, and work, while EQ-5D-5L better reflects physical health changes. Test–retest reliability was fair to good for EQ-HWB-S dimensions and moderate to good for EQ-5D-5L, with good reliability for index scores and EQ VAS (ICC: 0.70–0.77). Both tools detected improvement as children recovered, with EQ-5D-5L showing slightly higher responsiveness (effect size: 0.77–0.87) than EQ-HWB-S (0.62–0.74). |
| **Comparisons among disease-specific measures** | | | | | |
| Burks-2021 (43) | Dementia | HRQOL/Wellbeing impact | - QOL-AD - DEMQOL - ADRQL - QUALIDEM - QUALID - Some tools used for both self-report and proxy ratings | Self-rated versus proxy-rated QOL scores | Self-reported QOL ratings is generally higher than proxy-rated QOL, with depression, unmet needs, functional impairment, and caregiver burden lowering scores. Using both self- and proxy-reports provides a more balanced view, as proxies may overemphasize negatives and under-recognize issues like pain. People with dementia can reliably report their own QOL, even with cognitive decline. |
| Messina-2019 (44) | Spinal muscular atrophy (SMA) | Caregiver burden | 36 measures identified§ | - Primary focus (HRQOL, ADL, or caregiver burden, or a combination). - Suitability for different SMA types 1, 2, or 3 and specific age groups - SMA-specific or more broadly for neuromuscular disorders - Used and validated in SMA cohorts - If reliability and validity data were available, including use in longitudinal studies. - Undergone Rasch analysis | The review identified 36 tools assessing QOL, ADLs, and caregiver burden in SMA, with only 6 specifically designed for SMA and the rest for broader neuromuscular conditions. Tools were categorised by focus: 12 for HRQOL, 5 for ADLs, 9 for caregiver burden, and 10 with combined items. The review also noted which tools underwent Rasch analysis and their suitability across SMA types and age groups. |
| Sarri-2018 (45) | Sickle cell disease | Not applicable | Identified 24 patient-reported outcome instruments. | - Reliability (internal consistency, test-retest)  - Validity (content validity, construct validity)  - Responsiveness. | No validated patient-reported outcome instruments specifically designed to measure the burden on caregivers of children with sickle cell disease were identified. |
| Thomas-2019 (46) | Alzheimer’s disease | Caregiver burden | - Objective measures (from EVALUATE-AD): Continuous monitoring of caregiving activities, physical capacity/mobility, sleep patterns, cognitive functions, and physiological health - Subjective measures (from Tele-STAR): FAQ, NPI, ZBI-12. | Objective measures (step counts, sleep duration) versus caregivers’ subjective reports of care recipient behaviours and burden | Although the caregiver’s self-reported burden remained stable despite increasing patient behavioural challenges, objective monitoring showed reduced activity and more time spent alone, suggesting that objective measures can reveal subtle impacts on wellbeing not captured by subjective reports. |

*ADRQL, Alzheimer's Disease-Related Quality of Life; AQOL-8D, Assessment of Quality of Life 8 Dimensions; ASCOT-Carer, Adult Social Care Outcomes Toolkit for Carers; AUC, Area under the receiver operating characteristic (curve);*

*BDI,* Beck Depression Inventory; *BRS, Brief Resilience Scale; BSFC, Burden Scale for Family Caregivers;*

*CarGOQOL, CareGiver Oncology Quality of Life questionnaire; Carer-QOL, Care-related Quality of Life instrument; CBI, Caregiver Burden Inventory; CDS, Caregiver Distress Scale; CES, Carer Experience Scale; CES-D, Center for Epidemiologic Studies Depression Scale; CFA, Confirmatory Factor Analysis; CHD, Congenital heart disease; COPD, Chronic obstructive pulmonary disease; CRA, Caregiver Reaction Assessment; CR-SPB, Care Recipient Self-Perceived Burden;*

*DEMQOL, Dementia Quality of Life instrument; DRS, Dyadic Relationship Scale;*

*EFA, Exploratory Factor Analysis; EORTC QLQ-C30, European Organisation for Research and Treatment of Cancer Quality of Life Group Core Questionnaire; EQ-5D, EuroQol health-related quality of life 5 Dimensions; EQ-5D-Y-3L, EuroQol health-related quality of life of children and adolescents; EQ-HWB(-S), EQ Health and Wellbeing (Short) instrument; EQ-TIPS, EuroQol Toddler and Infant Populations; EQ VAS, EuroQol visual analogue scale;*

*FAQ, Functional Activities Questionnaire; FCR, Family Caregiving Role;*

*GHQ-28, General Health Questionnaire 28-item version;*

*HADS, Hospital Anxiety and Depression Scale; HRQOL, Health-related quality of life; HUI-3, Health Utilities Index-Mark*III;

[*(I)*](https://pubmed.ncbi.nlm.nih.gov/12818023/)*ADLs, (Instrumental) Activities of Daily Living; ICC, Intraclass correlation coefficient; ICECAP-A, ICEpop CAPability measure for Adults; IEQ, Involvement Evaluation Questionnaire; IOFS, Impact On Family Scale;*

*J-WFES, Japanese Work-Family Enrichment Scale;*

*NPI, Neuropsychiatric Inventory;*

*OHA, Overall Health Assessment (OHA);*

*PedsQL, Pediatric Quality of Life inventory; Proxy-CB, Proxy Assessment of Caregiver Burden;*

*QOL-AD, Quality of Life in Alzheimer's Disease scale; QUALID, Quality of Life in Late-stage Dementia; QUALIDEM, Quality of Life for People with Dementia*

*Rel.SS, Relatives' Stress Scale; ROC, Receiver operating characteristic (curve); RSS, Relationship Satisfaction Scale; RUD, Resource Utilisation in Dementia;*

*SCB, Screen for Caregiver Burden; SCD, Spinal cord injury/damage; SEIQOL-DW, Schedule for Evaluation of Individual Quality of Life – Direct Weighting; SF-36, Short Form 36 items; SMA, Spinal muscular atrophy; SPB, Self-Perceived Burden;* STAI, State-Trait Anxiety Inventory; *SWEMWBS, Short Warwick-Edinburgh Mental Wellbeing Scale;*

*WFES-PN and FWES-PN, Work-Family Enrichment Scale for Parent Nurses;*

*ZBI, Zarit Burden Interview*

** Nine main instruments in dermatology: Family Dermatology Life Quality Index (FDLQI), Dermatitis Family Index (DFI), Parents' Index of Quality of Life in Atopic Dermatitis (PIQoL-AD), Measure of quality of life in primary caregivers of children with atopic dermatitis (QPCAD), Childhood Atopic Dermatitis Impact Scale (CADIS), Psoriasis Family Index (PFI), FamilyPso, Epidermolysis Bullosa Burden of Disease (EB-BoD), and Family Burden Ichthyosis (FBI).*

*† Reported measures:*

- *Spasticity-specific patient-reported outcome measures: Patient Reported Impact of Spasticity Measure (PRISM) and Spinal Cord Injury-Spasticity Evaluation Tool (SCI-SET)*
- *Generic HRQOL measures: SF-36 and its modifications (SF-36V, SF-36E, SF-36 WW), Life Situation Questionnaire Revised (LSQ-R), Quality of Life Index SCI version (QLI-SCI), Sickness Impact Profile (SIP68), World Health Organization Quality of Life-BREF scale (WHOQOL-BREF)*
- *Preference-based measures: HUI, SF-6D, EQ-5D, EQ-5D-3L, EQ-5D-5L, Quality of Wellbeing Self-Administered (QWB-SA), AQOL-8D*
- *Caregiver burden measures: CBS, ZBI*

*§ 36 measures identified, specific examples mentioned including: SMA Health Index (SMA HI), SMA Independence Scale (SMAIS), Patient Reported Impact of Symptoms in Spinal Muscular Atrophy (PRISM-SMA), Work Activity Work Productivity and Activity Impairment (WPAI), Infant Toddler Quality of Life Questionnaire (ITQOL47), Egen Klassifikation 2 Scale (EK2), Pediatric Evaluation of Disability Inventory Computer-Adaptive Test (PEDI-CAT), Pediatric Quality of Life Inventory generic core (PedsQL), and Quality of Life in Neuromuscular Diseases (QOL–NMD).*

**Table S4.3. Time Trade-Off studies for utility elicitation.**

| **Study** | **Care recipient condition** | **Methodology notes** | **Patient TTO utility range** | **Caregiver TTO utility range** |
| --- | --- | --- | --- | --- |
| Aggio-2024 (125) | Alopecia areata | (1) 1 caregiver, 5 patient vignettes were developed based on literature review, clinical trial data, qualitative interviews with patients, caregivers, and clinical experts; (2) TTO + VAS + EQ-5D-5L interviews with members of the general public for utility value elicitation. | 0.50 – 0.92 (hair loss severity-specific) | 0.88 |
| Lo-2021 (135) | Lennox-Gastaut syndrome | (1) 24 vignettes were developed based on literature review, qualitative interviews with caregivers and clinical experts (varied by the number of seizures and seizure-free days per month); (2) TTO + VAS interviews with members of the general public for utility value elicitation. | −0.19 – 0.75 (seizure-specific) | 0.03 – 0.81 |
|  | Dravet syndrome | As above | 0.17 – 0.78  (seizure-specific) | 0.51 – 0.88 |
| Lo-2021 (134) | Hereditary angioedema | (1) Vignettes were developed based on qualitative interviews with patients, caregivers, clinical experts; (2) TTO + VAS interviews with members of the general public for utility value elicitation. | 0.13 – 0.78 (swelling attack-specific) | 0.76 |
| Lo-2022 (136) | Tuberous sclerosis complex | (1) 16 vignettes were developed based on literature review, qualitative interviews with caregivers and healthcare professionals; (2) TTO + VAS interviews with members of the general public for utility value elicitation. | −0.23 – 0.73  (seizure-specific) | 0.22 – 0.91 |
| Simon-2019 (138) | Infantile diseases (Krabbe, phenylketonuria, and Pompe) | (1) 18 health state (4 phenylketonuria, 6 Krabbe, 6 Pompe, and 2 enzyme replacement therapy) were developed in consultation with patients and medical experts; (2) TTO interviews with adult patients and parents for utility value elicitation. | 0.37 – 0.80 (advanced stages) | 0.07 - 0.19 (parental spillover) |
| Williams-2020 (140) | Cutaneous T-cell lymphoma | (1) 4 caregiver vignettes were developed based on literature review and interviews with specialists; (2) TTO + VAS + EQ-5D-5L interviews with members of the general public for utility value elicitation. | Not applicable | 0.37 – 0.52 |

*HRQOL, Health-related quality of life; TTO, Time trade-off; VAS, Visual analogue scale.*

**Table S4.4. Mediators and moderators of spillover effects.**

| **Study** | **Care recipient condition** | **Spillover domain** | **Conceptual framework for analysing mediating/moderating effects** | **Independent variables** | **Mediating/Moderating variables** | **Outcome variables** | **Findings** |
| --- | --- | --- | --- | --- | --- | --- | --- |
| **Mediators** | | | | | | | |
| Abed Al Wahad-2024 (47) | Cancer | Caregiver burden  HRQOL/Wellbeing impact | Not specified | Medical visits, patient self-rated health, patient depressive symptoms | Caregiver burden | Caregiver depressive symptoms | 80% caregivers experienced psychological burden and 50% reported depressive symptoms, with higher burden linked to female caregivers and patients who were less educated, less healthy, or more depressed. Caregiver burden **fully mediated** the effects of medical visit frequency and patient self-rated health on depressive symptoms. |
| Barber-2025 (48) | IgE-mediated food allergy | Caregiver burden | Lazarus and Folkman's (1984) Transactional Model of Stress and Coping | Perceived food allergy severity | Caregiver food allergy self-efficacy | Caregiver food allergy burden | Caregiver food allergy self-efficacy **partially mediated** the relationship between perceived food allergy severity and caregiver burden. Higher perceived severity was associated with lower self-efficacy and greater burden, whereas greater self-efficacy was linked to reduced burden (β = 0.16, SE = 0.07, CI 0.04–0.33). |
| Borchet-2021 (49) | Multiple diseases | HRQOL/Wellbeing impact | Not specified | Types of parentification | QOL | Academic achievement | Of **six mediation models** tested, two—instrumental parentification toward parents and siblings—showed good fit and interpretability. Instrumental parentification was positively associated with school achievement, and this relationship was mediated by adolescents’ general quality of life, suggesting that parentification may foster skills such as task management, coping strategies, and self-efficacy that benefit academic performance. |
| Cenkçi-2024 (50) | Cancer | Caregiver burden HRQOL/Wellbeing impact | Baron (1986)'s conceptual model of mediator/moderator effect (mentioned) | Stress, anxiety, and depression | Perceived social support | Caregiver burden | Caregiving burden, depression, anxiety, stress, and perceived social support were interrelated, and analyses of six models indicated that perceived social support **mediated**—but did not moderate—the relationships between stress and caregiving burden, depression, and anxiety. |
| Guan-2023 (51) | Schizophrenia | Caregiver burden HRQOL/Wellbeing impact | Folkman’s (2008) stress and coping model | Positive aspects of caregiving | Hope Social support | Caregiving burden and positive aspects of caregiving | Caregiving burden was negatively associated with positive aspects of caregiving, with hope and social support **mediating** this relationship; serial mediation indicated that higher burden reduced hope, which in turn diminished social support, leading to fewer positive caregiving experiences. |
| Handayani-2024 (52) | Stroke | Caregiver burden HRQOL/Wellbeing impact | Not specified | Caregiver burden | Resilience | Caregiver QOL | Resilience **partially mediated** the impact of caregiver burden on QOL (indirect effect = −0.042; direct effect = −0.321), with burden negatively associated with resilience (β = −0.279, R² = 0.172) and QOL (β = −0.279), and resilience positively predicting QoL (β = 0.152). Burden and resilience explained 34.7% of the variance in QOL (p < 0.01). |
| Ravyts-2024 (53) | Multiple diseases (physical, psychological, cognitive, developmental, or age-related impairments) | Caregiver burden HRQOL/Wellbeing impact | Not specified | Sleep disturbance | Mental health symptoms (anxiety and depression) | Caregiver QOL | Sleep disturbance indirectly reduced quality of life through increased mental health symptoms (β = −0.21, *p* = 0.001), with this effect amplified under higher caregiving demands (β = 0.33, *p* = 0.002). |
| Tsai-2018 (54) | Stroke | Caregiver burden HRQOL/Wellbeing impact | Not specified | Patient characteristics, caregiver characteristics, family resources | Caregiver burden | Caregiver QOL | Lower caregiver QOL was predicted by higher burden, lower education, poorer self-rated health, lower family income, and spouses covering medical fees, with poor health and low income being the strongest predictors. Financial factors directly reduced QOL, while the effects of lower education and poor/fair health were **partially mediated** by caregiver burden. |
| Vrettos-2023 (55) | Old age (individuals older than 65) | HRQOL/Wellbeing impact | White et al.’s caregiving model and Tsai et al.’s mediation model | Patient frailty | Caregivers’ subjective burden | Caregiver HRQOL | Caregivers’ subjective burden was negatively associated with both physical and mental HRQOL and **largely mediated** the effects of socio-demographics, caregiving duration, and patient frailty and comorbidities on caregiver HRQOL. |
| Wang-2025 (56) | Severe mental illnesses | Caregiver burden | Stress-buffering model | Patient HRQOL | Caregivers’ social support | Family burden | Caregivers’ social support was negatively correlated with family burden and **partially mediated** the relationship between patients’ HRQOL and family burden (*r* = −0.54, β = −0.016, mediating effect = 2.75%, p < 0.01). |
| Yu-2020 (57) | Schizophrenia | Caregiver burden HRQOL/Wellbeing impact | Pearlin’s stress process model | People living with schizophrenia global functioning | Caregiver appraisals (subjective burden, family functioning, caregiving rewarding feelings) | Caregiver depression | Caregiver perceived burden, family functioning, and positive caregiving feelings mediated the relationship between patient global functioning and caregiver depression, with perceived burden having the strongest effect. The stress process differed by caregiver type: perceived burden mediated outcomes for both parents and spouses, while family functioning mediated only parent depressive symptoms. |
| **Moderators** | | | | | | | |
| Diederich-2020 (58) | Old age | Willingness to provide caregiving over employment | Interaction terms (country of birth x birth cohort; country of birth x survey wave) | Birth cohort (age at reunification) | - Country of birth (East vs. West Germany) - Survey wave (time) | Valuation of informal caregiving relative to employment | People who grew up in East Germany place a higher value on caring for elderly parents than those who grew up in West Germany. This difference is strongest among older cohorts and has not significantly changed even 20 years after reunification. |
| La-2024 (59) | Cancer | Caregiver burden HRQOL/Wellbeing impact | Adapted from the Cancer Family Caregiving Experience Model | Caregiver burden | Spirituality | Caregiver depression | Spirituality protected caregivers against burden, **moderating** the relationship between depression and caregiver burden over time (*b* = −1.35, *p* = 0.015), with lower spirituality strengthening this link, particularly for schedule, financial, and family-support burdens. |
| Lovén-2017 (60) | Diabetes | Productivity loss | Framework developed by Bolin et al. (2002) and Jacobson (2000) | Sibling of a child with diabetes | Sibling gender and age at which the diabetic sibling was diagnosed | Sibling annual labour earnings | Mediation analyses showed that education, marital status, and having children did not explain the differences, and early childhood ability was ruled out. Time-invariant traits such as ability and resilience **moderated** the spillover effect, suggesting high-ability individuals may offset the earnings penalty. |
| Newmyer-2023 (61) | Old age (individuals older than 50) | HRQOL/Wellbeing impact | “Retired Husband Syndrome” framework and the family stress model | Spouse’s retirement status | Spouse’s gender and spouse's own retirement status | Physical health (ADLs, IADLs, obesity) and mental health (depressive symptoms) | Men, especially those not working, experienced the greatest negative health spillovers from a spouse’s retirement, particularly in physical health, whereas women were largely unaffected. A family stress model may better capture gender differences in health effects than the Retired Husband Syndrome framework. |
| Oh-2024 (62) | Old age | Informal care time | Not specified | Care-related characteristics (satisfaction, perceived stress, care attitude, and physical strain) | Care time (daily average hours spent on caregiving) | Caregiver burden | Caregiver burden was higher among informal caregivers, with stress, physical strain, and care time increasing risk in both groups. Care time moderated the relationship between care attitude and burden, such that attitudes were significantly associated with burden when care time was 1 SD above the mean (b = −0.903, SE = 0.106, p < 0.001), among formal caregivers only. |
| Orford-2019 (63) | Alcohol/drug/substance misuse | Caregiver burden HRQOL/Wellbeing impact | Stress–Strain–Coping–Support model | Family Member Impact | Coping strategies | Symptoms | Moderation analyses showed coping strategies (engagement and tolerance) significantly influenced the strength of this association, with small but meaningful interaction effects that added explanatory power to the regression. |
| Sun-2024 (64) | Cancer | Caregiver burden | Pearlin’s stress process model | Caregiving factors (caregiver commitment, time demand, number of close companions, emotional/informational support) | Family relationship quality | Caregiver burden | Caregiver burden increased over time, but supportive family relationships mitigated this rise, whereas a stronger sense of caregiving commitment was associated with greater burden. |
| Xu-2021 (65) | Dementia | Caregiver burden | Pearlin’s stress process model | Caregiving intensity | Social support (network size, received support, satisfaction with support, negative interactions) | Caregiver burden | Social support, including received support, satisfaction, and network size, **buffered** the impact of caregiving hours on burden, with social network showing the strongest effect when indicators were examined together, although negative interactions directly increased burden. |
| Zhou-2024 (66) | Dementia | Caregiver burden | Stress-buffering hypothesis | Caregiving intensity | Home-based care services (referral, household care, skilled care, mental healthcare) | Caregiver burden | Home-based care services did not directly predict burden but **moderated** the effects of caregiving tasks, with referral services uniquely buffering the impact of supervision time. |

*(I)ADL, (Instrumental) activities of daily living; CI, Confidence interval; HRQOL, Health-related quality of life; SD, Standard deviation; SE, Standard error.*

**Table S4.5. Factors acting as both mediators and moderators.**

| **Study** | **Care recipient condition** | **Spillover domain** | **Conceptual framework for analysing mediating and moderating effects** | **Independent variables** | **Mediating variables** | **Moderating variables** | **Outcome variables** | **Findings** |
| --- | --- | --- | --- | --- | --- | --- | --- | --- |
| Anderson-2020 (67) | Traumatic brain injury | Caregiver burden HRQOL/Wellbeing impact | Model of resilience in family caregivers of individuals with traumatic brain injury | Resilience | Hope Social support | Positive mental health Resilience combined with hope | Caregiver burden Mental health Negative affect | Caregiver resilience in traumatic brain injury is strongly influenced by self-efficacy and social support, which in turn reduce burden, while positive emotions enhance resilience and negative emotions increase burden. |
| Horváth-2019 (68) | Alcohol misuse | Caregiver burden  HRQOL/Wellbeing impact | Stress-Strain-Coping-Support model | Stress | - Burden - Tolerant-inactive coping | Coping | Psychological strain (e.g., hopelessness, anxiety, depression, anger) and physical symptoms. | Findings supported the moderating role of engaged coping and the additive effect of tolerant-inactive coping and burden on hopelessness. Mediation analyses showed significant indirect pathways linking perceived alcohol-related problems to hopelessness via burden, tolerant coping, or their sequential combination, consistent with the Stress-Strain-Coping-Support model’s suggestion. |
| Kim-2022 (69) | Cancer | Caregiver burden HRQOL/Wellbeing impact | Stress process model | Caregiver depression | Caregiver burden | Spousal relationship | Caregiver QOL | Caregiver depression reduced quality of life, partially mediated by increased burden, while stronger spousal relationships buffered the negative impact of burden on QOL. |
| López-Martínez-2024 (70) | Old age | Caregiver burden HRQOL/Wellbeing impact | Not specified | Coping strategies | Subjective caregiver burden | Subjective caregiver burden | Anxiety symptoms | Subjective caregiver burden both mediated and moderated the impact of coping strategies on anxiety symptoms. Anxiety was positively associated with proactive coping (B = 0.13), planning (B = 0.15), self-distraction (B = 0.24), denial (B = 1.15), venting (B = 0.94), and self-blame (B = 0.90), and negatively associated with positive reframing (B = −0.83) and acceptance (B = −0.75), with burden mediating these relationships and moderating the effects of planning and denial. |

*HRQOL, Health-related quality of life.*

**References**

1. Al-Janabi H, Efstathiou N, McLoughlin C, Calvert M, Oyebode J. The scope of carer effects and their inclusion in decision-making: A UK-based Delphi study. BMC Health Serv Res. 2021;21(1):752.

2. Angelini V, Costa-Font J. Health and wellbeing spillovers of a partner's cancer diagnosis. Journal of Economic Behavior & Organization. 2023;212:422-37.

3. Aranda-Reneo I, Rodríguez-Sánchez B, Peña-Longobardo LM, Oliva-Moreno J, López-Bastida J. Can the consideration of societal costs change the recommendation of economic evaluations in the field of rare diseases? An empirical analysis. Value Health. 2021;24(3):431-42.

4. Duevel JA, Hasemann L, Pena-Longobardo LM, Rodriguez-Sanchez B, Aranda-Reneo I, Oliva-Moreno J, et al. Considering the societal perspective in economic evaluations: a systematic review in the case of depression. Health Econ Rev. 2020;10(1):32.

5. Lavelle TA, D'Cruz BN, Mohit B, Ungar WJ, Prosser LA, Tsiplova K. Family spillover effects in pediatric cost-utility analyses. Applied Health Economics and Health Policy. 2019;17(2):163-74.

6. Leech AA, Lin P-J, D'Cruz B, Parsons SK, Lavelle TA. Family spillover effects: Are economic evaluations misrepresenting the value of healthcare interventions to society? Applied Health Economics and Health Policy. 2023;21(1):5-10.

7. Lin PJ, D'Cruz B, Leech AA, Neumann PJ, Sanon Aigbogun M, Oberdhan D, Lavelle TA. Family and caregiver spillover effects in cost-utility analyses of Alzheimer's Disease interventions. Pharmacoeconomics. 2019;37(4):597-608.

8. Ma S, Olchanski N, Cohen JT, Ollendorf DA, Neumann PJ, Kim DD. The impact of broader value elements on cost-effectiveness analysis: Two case studies. Value Health. 2022;25(8):1336-43.

9. Pennington BM. Inclusion of carer health-related quality of life in National Institute for Health and Care Excellence appraisals. Value Health. 2020;23(10):1349-57.

10. Ride J. Setting the boundaries for economic evaluation: Investigating time horizon and family effects in the case of postnatal depression. Value Health. 2018;21(5):573-80.

11. Rodriguez-Sanchez B, Aranda-Reneo I, Oliva-Moreno J, Lopez-Bastida J. Assessing the effect of including social costs in economic evaluations of diabetes-related interventions: A systematic review. Clinicoecon Outcomes Res. 2021;13:307-34.

12. Rodríguez-Sánchez B, Daugbjerg S, Peña-Longobardo LM, Oliva-Moreno J, Aranda-Reneo I, Cicchetti A, López-Bastida J. Does the inclusion of societal costs change the economic evaluations recommendations? A systematic review for multiple sclerosis disease. Eur J Health Econ. 2023;24(2):247-77.

13. Yuasa A, Yonemoto N, Kamei K, Murofushi T, LoPresti M, Taneja A, et al. Systematic literature review of the use of productivity losses/gains in cost-effectiveness analyses of immune-mediated disorders. Adv Ther. 2022;39(12):5327-50.

14. Bhadhuri A, Al-Janabi H, Jowett S, Jolly K. Incorporating household spillovers in cost utility analysis: A case study using behavior change in COPD. Int J Technol Assess Health Care. 2019;35(3):212-20.

15. de Groot S, Santi I, Bakx P, Wouterse B, van Baal P. Informal care costs according to age and proximity to death to support cost-effectiveness analyses. Pharmacoeconomics. 2023.

16. Mott DJ, Schirrmacher H, Al-Janabi H, Guest S, Pennington B, Scheuer N, et al. Modelling Spillover Effects on Informal Carers: The Carer QALY Trap. Pharmacoeconomics. 2023;41(12):1557-61.

17. Shafrin J, Dennen S, Pednekar P, Birch K, Bhor M, Kanter J, Neumann P. For which diseases do broader value elements matter most? An evaluation across 20 ICER evidence reports. J Manag Care Spec Pharm. 2021;27(5):650-9.

18. Breslau RM, Cohen JT, Diaz J, Malcolm B, Neumann PJ. A review of HTA guidelines on societal and novel value elements. Int J Technol Assess Health Care. 2023;39(1):e31.

19. Canadian Agency for Drugs and Technologies in Health. Measuring and Valuing Health for Children: A Review of the Evidence. Canadian journal of health technologies. 2024;4(9).

20. Drost RMWA, van der Putten IM, Ruwaard D, Evers SMAA, Paulus ATG. Conceptualizations of the societal perspective within economic evaluations: A systematic review. International Journal of Technology Assessment in Health Care. 2017;33(1):251-60.

21. Park JY, Marcum ZA, Garrison LP. Toward a broader concept of societal value: family spillovers in Alzheimer's disease. International Journal of Technology Assessment in Health Care. 2022;38(1):1-5.

22. Bucholc J, McCaffrey N, Ugalde A, Muldowney A, Rand S, Hoefman R, et al. How well do the adult social care outcomes toolkit for carers, carer experience scale and care-related quality of life capture aspects of quality of life important to informal carers in Australia? Qual Life Res. 2023;32(11):3109-21.

23. Eagleson KJ, McCombs D, Gerlich TM, Justo RN, Kasparian NA, Bora S. Systematic Review of Instruments Assessing Psychosocial Adaptation and Outcomes Among Families of Children With Congenital Heart Disease. J Pediatr Psychol. 2023;48(6):537-52.

24. Engel L, Rand S, Hoefman R, Bucholc J, Mihalopoulos C, Muldowney A, et al. Measuring carer outcomes in an economic evaluation: a content comparison of the Adult Social Care Outcomes Toolkit for Carers, Carer Experience Scale, and Care-Related Quality of Life using exploratory factor analysis. Med Decis Making. 2020;40(7):885-96.

25. Gonçalves-Pereira M, González-Fraile E, Santos-Zorrozúa B, Martín-Carrasco M, Fernández-Catalina P, Domínguez-Panchón AI, et al. Assessment of the consequences of caregiving in psychosis: a psychometric comparison of the Zarit Burden Interview (ZBI) and the Involvement Evaluation Questionnaire (IEQ). Health Qual Life Outcomes. 2017;15(1):63.

26. Hamidou Z, Baumstarck K, Chinot O, Barlesi F, Salas S, Leroy T, Auquier P. Domains of quality of life freely expressed by cancer patients and their caregivers: Contribution of the SEIQoL. Health and Quality of Life Outcomes. 2017;15.

27. Kawakita T, Hosoda Y. Work–family enrichment among parent nurses: a cross-sectional scale development and validation study. BMC Nursing. 2024;23(1):665.

28. Kudra A, Lees C, Morrell-Scott N. Measuring carer burden in informal carers of patients with long-term conditions. Br J Community Nurs. 2017;22(5):230-6.

29. Kuharic M, Mulhern B, Sharp LK, Turpin RS, Pickard AS. Delineating Care Recipient Burden Constructs: Development and Validation of the CARE-2B Scale for Care Recipient Self-Perceived Burden and Proxy Assessment of Caregiver Burden. Gerontologist. 2025;65(4).

30. McCaffrey N, Bucholc J, Rand S, Hoefman R, Ugalde A, Muldowney A, et al. Head-to-head comparison of the psychometric properties of 3 carer-related preference-based instruments. Value Health. 2020;23(11):1477-88.

31. Sampogna F, Finlay AY, Salek SS, Chernyshov P, Dalgard FJ, Evers AWM, et al. Measuring the impact of dermatological conditions on family and caregivers: A review of dermatology-specific instruments. J Eur Acad Dermatol Venereol. 2017;31(9):1429-39.

32. Tu JY, Jin G, Chen JH, Chen YC. Caregiver burden and dementia: A systematic review of self-report instruments. J Alzheimers Dis. 2022;86(4):1527-43.

33. Bhadhuri A, Jowett S, Jolly K, Al-Janabi H. A comparison of the validity and responsiveness of the EQ-5D-5L and SF-6D for measuring health spillovers: A study of the family impact of meningitis. Med Decis Making. 2017;37(8):882-93.

34. Brown CC, Tilford JM, Payakachat N, Williams DK, Kuhlthau KA, Pyne JM, et al. Measuring health spillover effects in caregivers of children with autism spectrum disorder: A comparison of the EQ-5D-3L and SF-6D. Pharmacoeconomics. 2019;37(4):609-20.

35. Ertzgaard P, Nene A, Kiekens C, Burns AS. A review and evaluation of patient-reported outcome measures for spasticity in persons with spinal cord damage: Recommendations from the Ability Network - an international initiative. J Spinal Cord Med. 2020;43(6):813-23.

36. Faraji H, Soleymani F, Yaseri M, Sahraian MA, Abdollahiasl A, Meftah A, Nikfar S. Choosing the Best Instrument for Measuring Health Spillover Effect in Caregivers of Patients With Multiple Sclerosis. Value in Health Regional Issues. 2024;39:49-56.

37. McLoughlin C, Goranitis I, Al-Janabi H. Validity and responsiveness of preference-based quality-of-life measures in informal carers: A comparison of 5 measures across 4 conditions. Value Health. 2020;23(6):782-90.

38. McLoughlin C, Goranitis I, Al-Janabi H. The Feasibility and Validity of Preference-Based Quality of Life Measures With Informal Carers: A Think-Aloud Study. Value Health. 2023;26(11):1655-64.

39. Monteiro AL, Kuharic M, Pickard AS. A comparison of a preliminary version of the EQ-HWB Short and the 5-level version EQ-5D. Value Health. 2022;25(4):534-43.

40. Reed C, Barrett A, Lebrec J, Dodel R, Jones RW, Vellas B, et al. How useful is the EQ-5D in assessing the impact of caring for people with Alzheimer's disease? Health Qual Life Outcomes. 2017;15(1):16.

41. Vatter S, McDonald KR, Stanmore E, McCormick SA, Clare L, Leroi I. A brief psychometric and clinimetric evaluation of self-report burden and mental health measures completed by care partners of people with Parkinson's-related dementia. Int Psychogeriatr. 2020;32(7):875-80.

42. Zhou W, Ding B, Busschbach J, Herdman M, Yang Z, Lu Y. EQ-5D-5L or EQ-HWB-S: Which is the Better Instrument for Capturing Spillover Effects in Parental Carers of Children with COVID-19? Pharmacoeconomics. 2025;43(5):555-67.

43. Burks HB, des Bordes JKA, Chadha R, Holmes HM, Rianon NJ. Quality of life assessment in older adults with dementia: A systematic review. Dementia and Geriatric Cognitive Disorders. 2021;50(2):103-10.

44. Messina S, Frongia AL, Antonaci L, Pera MC, Coratti G, Pane M, et al. A critical review of patient and parent caregiver oriented tools to assess health-related quality of life, activity of daily living and caregiver burden in spinal muscular atrophy. Neuromuscul Disord. 2019;29(12):940-50.

45. Sarri G, Bhor M, Abogunrin S, Farmer C, Nandal S, Halloway R, Revicki DA. Systematic literature review and assessment of patient-reported outcome instruments in sickle cell disease. Health and Quality of Life Outcomes. 2018;16.

46. Thomas NWD, Lindauer A, Kaye J. EVALUATE-AD and Tele-STAR: Novel methodologies for assessment of caregiver burden in a telehealth caregiver intervention - A case study. Dement Geriatr Cogn Disord. 2019;47(3):176-84.

47. Abed Al Wahad A, Elran-Barak R, Furer M, Abu Kamir G, Horowitz NA. Psychological burden and depressive symptoms in caregivers of hemato-oncological patients: the role of medical visits. Blood Adv. 2024;8(22):5917-24.

48. Barber M, Griffin D, Neshkes R, Kichline T, Sigel S, Herbert LJ. Caregiver food allergy self-efficacy explains the relationship between perceived food allergy severity and burden. Journal of Pediatric Psychology. 2025;50(3):289-96.

49. Borchet J, Lewandowska-Walter A, Połomski P, Peplińska A, Hooper LM. The relations among types of parentification, school achievement, and quality of life in early adolescence: An exploratory study. Frontiers in Psychology. 2021;12.

50. Cenkçi Ş, Çıvgın U, Yorulmaz E. The Role of Perceived Social Support on Stress, Anxiety, Depression and Care Burden Levels of Caregivers of Cancer Patients: A Turkish Republic of Northern Cyprus (TRNC) Example. Asian Pac J Cancer Prev. 2024;25(11):3865-76.

51. Guan Z, Huang C, Sun M, Bai X, Tang S. Caregiving burden and positive aspects of caregiving in schizophrenia: Mediating roles of hope and social support. Archives of Psychiatric Nursing. 2023;45:7-13.

52. Handayani F, Kusumaningrum NSD, Dwidiyanti M. The Correlation Between Caregivers Burden and Quality of Life Among Family Caregivers of Stroke Survivors: The Mediating Role of Resilience. Nursing: Research and Reviews. 2024;14(null):91-102.

53. Ravyts SG, Dzierzewski JM. Sleep Disturbance, Mental Health Symptoms, and Quality of Life: A Structural Equation Model Assessing Aspects of Caregiver Burden. Clin Gerontol. 2024;47(3):484-93.

54. Tsai YH, Lou MF, Feng TH, Chu TL, Chen YJ, Liu HE. Mediating effects of burden on quality of life for caregivers of first-time stroke patients discharged from the hospital within one year. BMC Neurol. 2018;18(1):50.

55. Vrettos I, Anagnostopoulos F, Voukelatou P, Panayiotou S, Kyvetos A, Nikas A, et al. Factors associated with health-related quality of life of informal caregivers of older patients and the mediating role of subjective caregivers' burden. Psychogeriatrics. 2023;23(2):286-97.

56. Wang S, Dong J, Wen L, Tang W, Zhang X, Fu J, et al. Relationship between quality of life of patients with severe mental illnesses and family burden of disease: the mediating effect of caregivers’ social support. BMC Public Health. 2025;25(1):616.

57. Yu Y, Liu ZW, Li TX, Li YL, Xiao SY, Tebes JK. Test of the stress process model of family caregivers of people living with schizophrenia in China. Soc Sci Med. 2020;259:113113.

58. Diederich F, König H-H, Brettschneider C. How politico-economic systems shape individuals’ value of elderly care: Evidence from the German reunification. The Gerontologist. 2020;60(2):350-8.

59. La IS, Johantgen M, Storr CL, Zhu S, Cagle JG, Ross A. Spirituality moderates the relationship between cancer caregiver burden and depression. Palliat Support Care. 2024;22(3):470-81.

60. Lovén I. Labor market consequences of growing up with a sibling with type 1-diabetes. Soc Sci Med. 2017;178:1-10.

61. Newmyer L, Lowrey KL, Levchenko Y. Unplanned costs and benefits: Gender and spousal spillover effects of retirement on health. Journal of Marriage and Family. 2023;85(5):1110-24.

62. Oh E, Moon S, Chung D, Choi R, Hong GS. The moderating effect of care time on care-related characteristics and caregiver burden: differences between formal and informal caregivers of dependent older adults. Front Public Health. 2024;12:1354263.

63. Orford J, Padin MdFR, Canfield M, Sakiyama HMT, Laranjeira R, Mitsuhiro SS. The burden experienced by Brazilian family members affected by their relatives’ alcohol or drug misuse. Drugs: Education, Prevention & Policy. 2019;26(2):157-65.

64. Sun R, Francis LE. Quality of Relationships and Caregiver Burden: A Longitudinal Study of Caregivers for Advanced Cancer Patients. J Gerontol B Psychol Sci Soc Sci. 2024;79(2).

65. Xu L, Liu Y, He H, Fields NL, Ivey DL, Kan C. Caregiving intensity and caregiver burden among caregivers of people with dementia: The moderating roles of social support. Arch Gerontol Geriatr. 2021;94:104334.

66. Zhou Y, Chan WC. Utilization of home-based care and its buffering effects between dementia caregiving intensity and caregiver burden in China. BMC Geriatr. 2024;24(1):913.

67. Anderson MI, Daher M, Simpson GK. A predictive model of resilience among family caregivers supporting relatives with traumatic brain injury (TBI): A structural equation modelling approach. Neuropsychol Rehabil. 2020;30(10):1925-46.

68. Horváth Z, Urbán R. Testing the stress-strain-coping-support (SSCS) model among family members of an alcohol misusing relative: The mediating effect of burden and tolerant-inactive coping. Addictive Behaviors. 2019;89:200-5.

69. Kim Y. The Impact of Depression on Quality of Life in Caregivers of Cancer Patients: A Moderated Mediation Model of Spousal Relationship and Caring Burden. Curr Oncol. 2022;29(11):8093-102.

70. López-Martínez C, Orgeta V, Frías-Osuna A, Del-Pino-Casado R. Coping and anxiety symptoms in family carers of dependent older people: Mediation and moderation effects of subjective caregiver burden. J Nurs Scholarsh. 2024;56(3):371-81.
