# Supplementary material for: Measuring and valuing spillover effects in caregivers and families: A scoping review
Source: PLoS One. 2026 Mar 24;21(3):e0337253. doi: 10.1371/journal.pone.0337253 (PMC13012466; doi:10.1371/journal.pone.0337253)
Supplement: S1 Data — (DOCX) [file pone.0337253.s005.docx]

**Selected studies**

Measuring and valuing spillover effects in caregivers and families: A scoping review.

| Reference number | Author (Year) | Access link |
| --- | --- | --- |
| [4] | Henry et al. (2024) | https://pubmed.ncbi.nlm.nih.gov/38041698/ |
| [12] | Grosse et al. (2019) | https://pubmed.ncbi.nlm.nih.gov/30953263/ |
| [13] | Wittenberg et al. (2019) | https://pubmed.ncbi.nlm.nih.gov/30887469/ |
| [16] | Al-Janabi et al. (2019) | https://www.sciencedirect.com/science/article/pii/S0277953619303673 |
| [18] | Tsai et al. (2018) | https://pubmed.ncbi.nlm.nih.gov/29699521/ |
| [23] | Campbell et al. (2024) | https://pubmed.ncbi.nlm.nih.gov/39213143/ |
| [31] | Angelini and Costa-Font (2023) | https://www.sciencedirect.com/science/article/pii/S016726812300207X |
| [32] | Al-Janabi et al. (2021) | https://link.springer.com/article/10.1186/s12913-021-06742-4 |
| [33] | Aranda-Reneo et al. (2021) | https://www.sciencedirect.com/science/article/pii/S1098301520344624 |
| [34] | Bannon et al. (2022) | https://pubmed.ncbi.nlm.nih.gov/35435998/ |
| [35] | Bhadhuri et al. (2019) | https://pubmed.ncbi.nlm.nih.gov/31064563/ |
| [36] | Bom et al. (2019) | https://www.sciencedirect.com/science/article/pii/S2212828X18301063 |
| [37] | Breslau et al. (2023) | https://pubmed.ncbi.nlm.nih.gov/37226807/ |
| [38] | Canadian Agency for Drugs and Technonogies in Health (2024) | https://www.ncbi.nlm.nih.gov/books/NBK608472/ |
| [39] | Canaway et al. (2019) | https://pubmed.ncbi.nlm.nih.gov/30877637/ |
| [40] | Das et al. (2024) | https://pubmed.ncbi.nlm.nih.gov/38977195/ |
| [41] | Daysal et al. (2022) | https://direct.mit.edu/rest/article/104/1/1/97714/Spillover-Effects-of-Early-Life-Medical |
| [42] | de Groot et al. (2023) | https://pubmed.ncbi.nlm.nih.gov/36725787/ |
| [43] | Dixon and Round (2019) | https://pubmed.ncbi.nlm.nih.gov/31104733/ |
| [44] | Drost et al. (2017) | https://pubmed.ncbi.nlm.nih.gov/28641592/ |
| [45] | Duevel et al. (2020) | https://pubmed.ncbi.nlm.nih.gov/32964372/ |
| [46] | Engel et al. (2021) | https://pubmed.ncbi.nlm.nih.gov/34423386/ |
| [47] | Fischer et al. (2024) | https://pubmed.ncbi.nlm.nih.gov/39224484/ |
| [48] | Fletcher and Marksteiner (2017) | https://www.aeaweb.org/articles?id=10.1257/pol.20150573 |
| [49] | Gardiner et al. (2017) | https://pubmed.ncbi.nlm.nih.gov/27670418/ |
| [50] | Handels et al. (2025) | https://pubmed.ncbi.nlm.nih.gov/39800459/ |
| [51] | Henry and Cullinan (2021) | https://www.sciencedirect.com/science/article/pii/S0277953621003282 |
| [52] | Henry and Cullinan (2024) | https://pubmed.ncbi.nlm.nih.gov/38185789/ |
| [53] | Henry and Cullinan (2025) | https://pubmed.ncbi.nlm.nih.gov/39395652/ |
| [54] | Igarashi and Ikeda (2022) | https://pubmed.ncbi.nlm.nih.gov/36039772/ |
| [55] | Kanters et al. (2024) | https://pubmed.ncbi.nlm.nih.gov/39377220/ |
| [56] | Lamsal et al. (2024) | https://pubmed.ncbi.nlm.nih.gov/38819718/ |
| [57] | Lamsal et al. (2024) | https://pubmed.ncbi.nlm.nih.gov/37945777/ |
| [58] | Lavelle et al. (2019) | https://pubmed.ncbi.nlm.nih.gov/30350218/ |
| [59] | Lee et al. (2022) | https://pubmed.ncbi.nlm.nih.gov/34378438/ |
| [60] | Leech et al. (2023) | https://pubmed.ncbi.nlm.nih.gov/35997896/ |
| [61] | Lin et al. (2019) | https://pubmed.ncbi.nlm.nih.gov/30903567/ |
| [62] | Lin et al. (2021) | https://pmc.ncbi.nlm.nih.gov/articles/PMC8559218/ |
| [63] | Liu et al. (2021) | https://pmc.ncbi.nlm.nih.gov/articles/PMC8252638/ |
| [64] | Ma et al. (2022) | https://pubmed.ncbi.nlm.nih.gov/35315331/ |
| [65] | Mattingly et al. (2022) | https://pubmed.ncbi.nlm.nih.gov/35607780/ |
| [66] | Mendoza-Jiménez et al. (2024) | https://pubmed.ncbi.nlm.nih.gov/38261132/ |
| [67] | Mott et al. (2023) | https://pubmed.ncbi.nlm.nih.gov/37659032/ |
| [68] | Muir and Keim-Malpass (2020) | https://pubmed.ncbi.nlm.nih.gov/32543221/ |
| [69] | Safrin et al. (2024) | https://pubmed.ncbi.nlm.nih.gov/39512185/ |
| [70] | Simoens et al. (2024) | https://pubmed.ncbi.nlm.nih.gov/39136368/ |
| [71] | Park et al. (2022) | https://pubmed.ncbi.nlm.nih.gov/36317687/ |
| [73] | Pennington (2020) | https://pubmed.ncbi.nlm.nih.gov/33032779/ |
| [74] | Pennington et al. (2025) | https://pubmed.ncbi.nlm.nih.gov/39343091/ |
| [75] | Phelps (2024) | https://pubmed.ncbi.nlm.nih.gov/38401798/ |
| [76] | Ride (2018) | https://pubmed.ncbi.nlm.nih.gov/29753355/ |
| [77] | Rodriguez-Sanchez et al. (2021) | https://pubmed.ncbi.nlm.nih.gov/33953579/ |
| [78] | Rodriguez-Sanchez et al. (2023) | https://pubmed.ncbi.nlm.nih.gov/35596098/ |
| [79] | Shafrin et al. (2021) | https://pubmed.ncbi.nlm.nih.gov/33779245/ |
| [80] | Schröder et al. (2023) | https://pubmed.ncbi.nlm.nih.gov/37068847/ |
| [81] | Urwin et al. (2021) | https://pubmed.ncbi.nlm.nih.gov/34324174/ |
| [82] | Urwin et al. (2021) | https://pubmed.ncbi.nlm.nih.gov/33845393/ |
| [83] | Urwin et al. (2021) | https://pmc.ncbi.nlm.nih.gov/articles/PMC8022080/ |
| [84] | Van Houtven et al. (2024) | https://pubmed.ncbi.nlm.nih.gov/39136596/ |
| [85] | Wu et al. (2020) | https://pubmed.ncbi.nlm.nih.gov/32266555/ |
| [86] | Yuasa et al. (2022) | https://pubmed.ncbi.nlm.nih.gov/36205907/ |
| [87] | Zang et al. (2023) | https://pmc.ncbi.nlm.nih.gov/articles/PMC10828899/ |
| [88] | Zhong et al. (2024) | https://pubmed.ncbi.nlm.nih.gov/38528583/ |
| [89] | Bhadhuri et al. (2017) | https://pubmed.ncbi.nlm.nih.gov/28525725/ |
| [90] | Brown et al. (2019) | https://pubmed.ncbi.nlm.nih.gov/30864066/ |
| [91] | Bucholc et al. (2023) | https://pubmed.ncbi.nlm.nih.gov/37356076/ |
| [92] | Burks et al. (2021) | https://pubmed.ncbi.nlm.nih.gov/34167127/ |
| [93] | Eagleson et al. (2023) | https://pubmed.ncbi.nlm.nih.gov/37221700/ |
| [94] | Engel et al. (2020) | https://pubmed.ncbi.nlm.nih.gov/32715900/ |
| [95] | Ertzgaard et al. (2020) | https://pubmed.ncbi.nlm.nih.gov/30758270/ |
| [96] | Faraji et al. (2024) | https://pubmed.ncbi.nlm.nih.gov/37979543/ |
| [97] | Gonçalves-Perei et al. (2017) | https://pubmed.ncbi.nlm.nih.gov/28381222/ |
| [98] | Hamidou et al. (2017) | https://pubmed.ncbi.nlm.nih.gov/28494773/ |
| [99] | Kawakita and Hosoda (2024) | https://pmc.ncbi.nlm.nih.gov/articles/PMC11411864/ |
| [100] | Kudra et al. (2017) | https://pubmed.ncbi.nlm.nih.gov/28467247/ |
| [101] | Kuharic et al. (2025) | https://pubmed.ncbi.nlm.nih.gov/39447028/ |
| [102] | McCaffrey et al. (2020) | https://pubmed.ncbi.nlm.nih.gov/33127019/ |
| [103] | McLoughlin et al. (2020) | https://pmc.ncbi.nlm.nih.gov/articles/PMC7532692/ |
| [104] | McLoughlin et al. (2023) | https://pubmed.ncbi.nlm.nih.gov/37516197/ |
| [105] | Messina et al. (2019) | https://pubmed.ncbi.nlm.nih.gov/31791871/ |
| [106] | Monteiro et al. (2022) | https://pubmed.ncbi.nlm.nih.gov/35279371/ |
| [107] | Reed et al. (2017) | https://pubmed.ncbi.nlm.nih.gov/28109287/ |
| [108] | Sampogna et al. (2017) | https://pubmed.ncbi.nlm.nih.gov/28426906/ |
| [109] | Sarri et al. (2018) | https://pubmed.ncbi.nlm.nih.gov/29784054/ |
| [110] | Thomas et al. (2019) | https://pmc.ncbi.nlm.nih.gov/articles/PMC8272983/ |
| [111] | Tu et al. (2022) | https://pubmed.ncbi.nlm.nih.gov/35253744/ |
| [112] | Vatter et al. (2020) | https://pubmed.ncbi.nlm.nih.gov/32744494/ |
| [113] | Zhou et al. (2025) | https://pubmed.ncbi.nlm.nih.gov/39907985/ |
| [114] | Elayan et al. (2024) | https://pubmed.ncbi.nlm.nih.gov/38294595/ |
| [115] | Engel et al. (2021) | https://pubmed.ncbi.nlm.nih.gov/34838285/ |
| [116] | Hanly et al. (2017) | https://pubmed.ncbi.nlm.nih.gov/28205149/ |
| [117] | Kanters et al. (2021) | https://pubmed.ncbi.nlm.nih.gov/33518030/ |
| [118] | Oliva-Moreno et al. (2019) | https://journals.plos.org/plosone/article?id=10.1371/journal.pone.0217016 |
| [119] | Ortega-Ortega et al. (2018) | https://www.scielosp.org/article/gs/2018.v32n5/411-417/ |
| [120] | Rabier et al. (2020) | https://pubmed.ncbi.nlm.nih.gov/32312345/ |
| [121] | Ramezani-Doroh et al. (2023) | https://pubmed.ncbi.nlm.nih.gov/37013619/ |
| [122] | Tubeuf et al. (2019) | https://pubmed.ncbi.nlm.nih.gov/30294758/ |
| [123] | Vilaplana-Prieto and  Oliva-Moreno (2025) | https://pubmed.ncbi.nlm.nih.gov/39117786/ |
| [124] | Aggio et al. (2024) | https://pubmed.ncbi.nlm.nih.gov/38551802/ |
| [125] | Al-Janabi et al. (2022) | https://pubmed.ncbi.nlm.nih.gov/34823129/ |
| [126] | Arora et al. (2019) | https://pubmed.ncbi.nlm.nih.gov/29512032/ |
| [127] | Cheneau and Rapp (2025) | https://pubmed.ncbi.nlm.nih.gov/39733835/ |
| [128] | Coe et al. (2018) | https://pubmed.ncbi.nlm.nih.gov/30222183/ |
| [129] | Costa-Font and Vilaplana‐Prieto (2025) | https://pubmed.ncbi.nlm.nih.gov/39888114/ |
| [130] | Engel et al. (2024) | https://pubmed.ncbi.nlm.nih.gov/38871025/ |
| [131] | Hoefman et al. (2019) | https://pubmed.ncbi.nlm.nih.gov/30298280/ |
| [132] | Jacobs et al. (2019) | https://pubmed.ncbi.nlm.nih.gov/30864064/ |
| [133] | Lo et al. (2022) | https://pubmed.ncbi.nlm.nih.gov/34532843/ |
| [134] | Lo et al. (2021) | https://pubmed.ncbi.nlm.nih.gov/34774333/ |
| [135] | Lo et al. (2022) | https://pubmed.ncbi.nlm.nih.gov/34524653/ |
| [136] | Mosquera and Rodríguez-Míguez (2018) | https://pubmed.ncbi.nlm.nih.gov/27749972/ |
| [137] | Simon et al. (2019) | https://pubmed.ncbi.nlm.nih.gov/30671727/ |
| [138] | Urwin et al. (2023) | https://www.sciencedirect.com/science/article/pii/S027795362300521X |
| [139] | Williams et al. (2020) | https://pubmed.ncbi.nlm.nih.gov/32644862/ |
| [140] | Al Wahad et al. (2024) | https://pubmed.ncbi.nlm.nih.gov/39348662/ |
| [141] | Barber et al. (2025) | https://pubmed.ncbi.nlm.nih.gov/39823372/ |
| [142] | Anderson et al. (2020) | https://pubmed.ncbi.nlm.nih.gov/31132931/ |
| [143] | Benjamin-Chung et al. (2017) | https://pubmed.ncbi.nlm.nih.gov/28449030/ |
| [144] | Borchet et al. (2021) | https://www.frontiersin.org/journals/psychology/articles/10.3389/fpsyg.2021.635171/full |
| [145] | Cenkçi et al. (2024) | https://pubmed.ncbi.nlm.nih.gov/39611910/ |
| [146] | Diederich et al. (2020) | https://pubmed.ncbi.nlm.nih.gov/31602474/ |
| [147] | Guan et al. (2023) | https://pubmed.ncbi.nlm.nih.gov/37544704/ |
| [148] | Handayani et al. (2024) | https://www.tandfonline.com/doi/full/10.2147/NRR.S435548 |
| [149] | Horváth and Urbán (2019) | https://www.sciencedirect.com/science/article/abs/pii/S030646031830724X |
| [150] | Kim (2022) | https://pubmed.ncbi.nlm.nih.gov/36354699/ |
| [151] | La et al. (2024) | https://pubmed.ncbi.nlm.nih.gov/38131143/ |
| [152] | López-Martínez et al. (2024) | https://pubmed.ncbi.nlm.nih.gov/38282022/ |
| [153] | Loven (2017) | https://pubmed.ncbi.nlm.nih.gov/28189818/ |
| [154] | Montoro-Gurich and Garcia-Vivar (2019) | https://pubmed.ncbi.nlm.nih.gov/30999801/ |
| [155] | Newmyer et al. (2023) | https://onlinelibrary.wiley.com/doi/10.1111/jomf.12925 |
| [156] | Oh et al. (2024) | https://pubmed.ncbi.nlm.nih.gov/38638476/ |
| [157] | Orford et al. (2019) | https://www.tandfonline.com/doi/full/10.1080/09687637.2017.1393500 |
| [158] | Ravyts and Dzierzewski (2024) | https://pubmed.ncbi.nlm.nih.gov/32597344/ |
| [159] | Sun and Francis (2024) | https://pubmed.ncbi.nlm.nih.gov/37889267/ |
| [160] | Vrettos et al. (2023) | https://pubmed.ncbi.nlm.nih.gov/36597270/ |
| [161] | Wang et al. (2025) | https://pubmed.ncbi.nlm.nih.gov/39953441/ |
| [162] | Wuttke-Linnemann et al. (2019) | https://pubmed.ncbi.nlm.nih.gov/30909228/ |
| [163] | Xu et al. (2021) | https://pubmed.ncbi.nlm.nih.gov/33516077/ |
| [164] | Yu et al. (2020) | https://pubmed.ncbi.nlm.nih.gov/32646627/ |
| [165] | Zhou and Chan (2024) | https://pubmed.ncbi.nlm.nih.gov/39501171/ |
| [169] | Peña-Longobardo and  Oliva-Moreno (2021) | https://www.ijhpm.com/article_4157.html |
