# Supplementary material for: Measuring and valuing spillover effects in caregivers and families: A scoping review
Source: PLoS One. 2026 Mar 24;21(3):e0337253. doi: 10.1371/journal.pone.0337253 (PMC13012466; doi:10.1371/journal.pone.0337253)
Supplement: Attachment — Submitted filename: Reviewer.doc [file pone.0337253.s006.doc]

**Reviewer**

**Measuring and valuing spillover effects in caregivers and families: A scoping review.**

Thank you for the opportunity to read and review this fascinating manuscript, which makes a substantial contribution to the academic literature. The article entitled *“Measuring and valuing spillover effects in caregivers and families: A scoping review”* is a high-quality scoping review with rigorous methodology and clear relevance to contemporary health systems. The topic is timely and addresses a critical shift in evaluation frameworks—from a patient-centric perspective to a broader societal perspective—by explicitly considering spillover effects on caregivers and families. This perspective is critical in the context of global population aging and the rising burden of chronic disease.

The manuscript makes a clear academic contribution to the fields of health economics, caregiving, long-term care, and health policy. A key strength of the review lies in its systematic examination of how spillover effects among caregivers and families can meaningfully influence cost-effectiveness outcomes and incremental cost-effectiveness ratios (ICERs). The authors convincingly demonstrate that failure to account for these effects may lead to policy decisions that do not fully reflect the actual societal costs and benefits of health interventions.

Methodologically, the review is conducted rigorously in accordance with established JBI and PRISMA-ScR frameworks and draws on a wide range of interdisciplinary databases spanning clinical, psychosocial, and health economic research. The manuscript serves as an effective mapping paper for the field. Significantly, the synthesis goes beyond descriptive reporting to include critical conceptual and methodological analysis, such as comparisons of generic, caregiver-specific, and disease-specific measurement instruments, as well as valuation approaches including opportunity cost, replacement cost, contingent valuation, and discrete choice experiments (DCEs). The discussion also addresses key methodological challenges, including double counting, equity bias, and family-size bias.

From a theoretical perspective, the manuscript offers significant value through its analysis of the mechanisms, mediators, and moderators of spillover effects. In particular, the emphasis on subjective caregiver burden as both an outcome and a mediating mechanism provides a strong conceptual bridge between health economics, nursing science, psychology, and family studies. This integrated perspective opens important avenues for future research, including survey development, longitudinal study designs, and intervention research targeting caregiver support.

In conclusion, spillover effects represent a critical yet often underappreciated component of health policy and health technology assessment. This manuscript not only synthesizes current knowledge but also provides clear directions for future research and policy development. As such, it constitutes an essential reference for the field and is highly suitable for publication in an international peer-reviewed journal.
